# Aberrant long-chain fatty acids metabolism and its interplay with immuno-inflammatory responses in relapsing-remitting multiple sclerosis

**DOI:** 10.3389/fimmu.2026.1766322

**Published:** 2026-03-24

**Authors:** Xiao-xi Zhu, Pei-juan Wang, He-xu Liu, Peng Sun, Li-mei Yu, Fan Yang

**Affiliations:** 1 Key Laboratory of Cell Engineering in Guizhou Province, Affiliated Hospital of Zunyi Medical University, Zunyi, China; 2 Department of Psychiatry, Nantong Fourth People’s Hospital, Nantong, China; 3 Department of Neurology, Affiliated Hospital of Zunyi Medical University, Zunyi, China; 4 Lishui Key Laboratory of Brain Health and Severe Brain Disorders. Lishui Second People’s Hospital, Wenzhou Medical University, Lishui, China; 5 Collaborative Innovation Center of Tissue Damage Repair and Regenerative Medicine of Ministry of Education, Zunyi Medical University, Zunyi, China; 6 Bio-X Institutes, Key Laboratory for the Genetics of Developmental and Neuropsychiatric Disorders, Ministry of Education, Shanghai Jiao Tong University, Shanghai, China

**Keywords:** chemokine, cytokine, immune-metabolic network, inflammation, long-chain fatty acid, mono-unsaturated fatty acid, poly-unsaturated fatty acid, relapsing-remitting multiple sclerosis

## Abstract

**Background:**

Growing evidence indicates significant alterations in fatty acid metabolism in patients with relapsing-remitting multiple sclerosis (RRMS). However, the metabolic status of long-chain fatty acids (LCFAs), including mono-unsaturated fatty acids (MUFAs) and poly-unsaturated fatty acids (PUFAs), and their potential link to immune-inflammatory responses during RRMS relapses, remain unclear. This study aims to uncover the aberrant metabolic signatures of LCFAs, potential LCFA biomarkers during RRMS relapses, and their interactive network with peripheral inflammatory responses.

**Methods:**

In this study, plasma samples from 20 RRMS patients and 22 age- and sex-matched healthy controls (HCs) were analyzed using liquid chromatography-tandem mass spectrometry (LC-MS/MS)-based untargeted metabolomics method.

**Results:**

Metabolomics analysis revealed marked changes in the LCFA metabolic profile of RRMS patients. Compared to HCs, 26 differentially abundant metabolites (DAMs) belonging to amino acids, fatty acids, and their derivatives were identified in RRMS samples, including significantly upregulated LCFA palmitic acid (FA 16:0) (*P_adj_* < 0.001), MUFA oleic acid (FA 18:1) (*P_adj_* < 0.05), PUFA arachidonic acid (FA 20:4) (*P_adj_* < 0.01), and significantly downregulated dodecanoic acid (FA 12:0) (*P_adj_* < 0.01). These DAMs were mainly enriched in amino acid, fatty acid, and lipid synthesis/metabolism pathways. Additionally, the circulating levels of pro-inflammatory factors TNF-α and IL17A were significantly elevated (*P_adj_* < 0.001), while the concentrations of chemokines such as IL1RA, CCL2, CCL3, CCL4, CCL5, PDGFB, IL7, CXCL8, IL9, and IL12A were significantly reduced (*P_adj_* < 0.01) in RRMS compared to HC samples. Linear regression analysis showed significant positive correlations between FA 20:4 and IL17A (r = 0.370, *P_adj_* < 0.05), and significant negative correlations between FA 16:0 and PDGFB (r = -0.339, *P_adj_* < 0.05). Receiver operating characteristic (ROC) curve analysis indicated that individual fatty acids (e.g., FA 12:0 [AUC = 0.881], FA 18:1 [AUC = 0.833], FA 16:0 [AUC = 0.881]) have high potential for predicting RRMS, with higher accuracy, specificity, and sensitivity when combining two (FA 12:0 and FA 18:1 [AUC = 0.929]) or four (FA 12:0, FA 18:1, FA 16:0, and FA 20:4 [AUC = 0.952]) fatty acids.

**Conclusion:**

Our results uncover the aberrant metabolic features of LCFAs and potential biomarkers in RRMS patients, and the interactive network and key molecular nodes between LCFAs and peripheral immune-inflammatory responses. The interplay between LCFAs and immuno-inflammation may drive the migration of inflammatory events from the periphery to the CNS, reigniting CNS neuroinflammation and causing RRMS relapses. These findings offer valuable insights for RRMS diagnosis and novel therapeutic development.

## 1 Introduction

Multiple sclerosis (MS), an autoimmune inflammatory disease, is caused by chronic axonal demyelination, which disrupts central nervous system (CNS) function and leads to severe disabilities like motor disorders, paralysis, autonomic dysfunction, and cognitive impairment (1–3). The MS prevalence has risen to 35.9 per 100,000 people (4), with 2.8 million affected individuals worldwide and approximately three quarters being women (4, 5). MS risk factors and associated factors involves genetic, epigenetic, and environmental factors (6–15), such as Epstein-Barr virus infection (16–18), low intake of vitamin D (19, 20), and smoking (21, 22). Emerging evidence implicates lipid-related factors in MS risk, including dietary saturated fat intake, obesity, metabolic syndrome, and altered gut microbiota affecting short-chain fatty acid production (23–27). These findings suggest that aberrant fatty acid/lipid metabolism is also a risk factor for MS. Based on clinical manifestations, MS is usually divided into different subtypes. The main subtype is relapsing-remitting MS (RRMS), constituting about 85% of cases, where patients experience at least two relapses (i.e., acute neurological dysfunction) and remissions (i.e., relative clinical stability) (1, 28), while primary progressive MS (PPMS) accounts for approximately 10%-15% of cases, with patients progressing from disease onset (29, 30). While traditional classifications distinguish RRMS from progressive forms, recent consensus frameworks emphasize MS as a biological continuum (31). However, for clinical trial and biomarker development purposes, the relapse-remitting phenotype remains a valid and important conceptual framework.

In recent decades, researchers have conducted numerous metabolome studies on MS patients, identifying differentially abundant metabolites (DAMs) related to MS (32–41). Genetic variations in lipid-metabolizing enzymes (e.g., *FADS* gene cluster polymorphisms) show significant frequency differences between Asian and Caucasian populations (42), potentially affecting PUFA metabolism (43). Additionally, gut microbiome composition differs across ethnic groups (44, 45), which may influence short-chain fatty acids (SCFAs) production (46). These biological factors, combined with underrepresentation of Asian cohorts in MS metabolomics literature (33), justify our focus on Chinese patients. Prior plasma lipidomic investigations in RRMS have revealed heterogeneous but reproducible alterations across distinct populations and disease stages. Shi et al. identified elevated lysophosphatidylcholines, sphingomyelins, and acylcarnitines in predominantly Western cohorts (47), while Penkert et al. reported decreased ether phospholipids (PC O-, PE O-) in German twins discordant for MS (48), and Schoeps et al. demonstrated that phosphatidylcholines predict neurofilament light chain levels in pediatric-onset disease (49). Notably, these studies focused largely on Caucasian populations with stable disease or mixed disease stages, leaving the metabolic signature of active relapse—particularly in Asian patients—underexplored. Amatruda et al. highlighted reduced lysophosphatidic acid 18:2 in progressive MS (50), suggesting lipid changes differ by disease phenotype, while Ladakis et al. showed brain lesion-specific sphingolipid dysregulation (51), implying peripheral lipid changes may reflect CNS pathology. However, the specific role of long-chain fatty acids (LCFAs, particularly PUFA and MUFA) during RRMS relapses and their interplay with peripheral immune responses remain uncharacterized, especially in Chinese patients whose genetic background and low-fat dietary patterns may yield distinct lipidomic profiles. Our study addresses these gaps by performing targeted LCFA analysis integrated with inflammatory mediator profiling in Chinese RRMS patients during acute relapse, establishing a relapse-specific LCFA-immune network and demonstrating superior biomarker accuracy through combinatorial LCFA models, thereby providing novel insights into the metabolic drivers of disease exacerbation in an underrepresented population. Additionally, our group previously reported altered plasma metabolic profiles in Chinese MS patients, identifying broad dysregulation across amino acids, lipids, and carbohydrates (33). However, that study had several limitations that the current investigation addresses: (1) It included mixed MS subtypes (RRMS and PPMS) and heterogeneous sampling (relapse and remission phases), precluding disease-stage specific interpretation; (2) It lacked systematic LCFA-immune correlation analysis; and (3) It did not specifically investigate LCFA metabolism as a mechanistic hypothesis. Critically, the 2021 study’s samples were not exclusively from relapses, making it impossible to distinguish relapse-specific metabolic signatures from chronic disease metabolism. The current manuscript represents a substantial advance by focusing specifically on LCFAs, their comprehensive immune network interactions, and relapse-phase pathophysiology in a treatment-naïve Chinese cohort.

To address these gaps, we performed LC-MS/MS-based untargeted metabolomics of plasma samples from Chinese RRMS patients during acute relapse. Plasma was selected over CSF or tissue because it provides a minimally invasive, clinically translatable matrix that captures systemic metabolic disturbances and peripheral immune activation central to MS pathogenesis. While our untargeted approach enabled unbiased discovery of novel metabolic networks, LCFAs emerged as a particularly dysregulated class, warranting detailed investigation of their interplay with inflammatory responses. we found significant changes in the metabolic profile of LCFAs and altered immune responses in peripheral system of RRMS-affected Chinese patients, where the PUFA FA 20:4 was modestly but significantly related to enhanced IL17A-mediated inflammatory responses. This may drive the migration of inflammation from the periphery to the CNS, thus activating neuroinflammation and promoting MS relapses. Our findings outlined an immunometabolic regulatory network of LCFAs and pro-inflammatory responses in the peripheral system of RRMS patients. This network may maintain the chronic inflammation status needed for MS development, and control CNS inflammation and RRMS relapse progression.

## 2 Materials and methods

### 2.1 Participant recruitment and biospecimen collection

A total of 20 patients with RRMS in the relapse period were diagnosed based on the 2017 McDonald criteria were recruited. Relapse was defined as: (1) Clinical criteria: New neurological symptoms lasting >24 hours, in the absence of fever or infection, with objective neurological examination findings by an attending neurologist; (2) Radiological criteria: Magnetic Resonance Imaging (MRI) performed within 7 days of symptom onset showing ≥1 new gadolinium-enhancing T1 lesion or new/enlarging T2 lesion compared to most recent prior scan. The symptom types of RRMS patients include optic neuritis (n = 6), motor deficits (n = 8), sensory deficits (n = 4), and brainstem/cerebellar symptoms (n = 2). Additionally, 22 age-, sex-, and body mass index (BMI)-matched healthy control (HC) subjects were simultaneously enrolled. Pearson chi-square test was used to analyze categorical variables (such as sex) between the two groups. Sample size calculation was based on a pilot study (n = 20) using G*Power method. Palmitic acid (FA 16:0), showing the largest effect size (Cohen’s d = 0.85), was selected as the primary endpoint for power analysis *a priori*. A minimum of 19 subjects per group was required for 80% power at α = 0.05 (two-tailed). We enrolled 20 RRMS patients and 22 HCs to account for potential missing data. *Post-hoc* sensitivity analysis confirmed 80% power to detect medium effects (d ≥ 0.65) for secondary metabolites. The demographic information (such as gender, age, and BMI) and clinical characteristics of all RRMS patients and HCs subjects were shown in the **Table 1**. The protocols of this study were reviewed and approved by the Ethics Committee of Affiliated Hospital of Zunyi Medical University (approval number KLL-2022-305), and the Ethics Committee of Lishui Second People’s Hospital (approval number 20171116-3) before participant enrollment. All subjects were fully informed and a written informed consent was obtained from each subject prior to recruitment. All RRMS patients were treatment-naïve at enrollment, having received no disease-modifying therapy (DMT, including traditional injectable medications such as Interferon-β and Glatiramer Acetate, first-line treatment drugs such as Fingolimod, Ocrelizumab, and Natalizumab, or immune-modulatory drugs such as Teriflunomide and Dimethyl Fumarate) since diagnosis. Due to non-compliance with the doctor’s instructions and self-discontinuation of medication, all RRMS patients had an untreated period of more than 20 months before being recruited. Prior to this, 20 RRMS patients received treatment with the drug Teriflunomide. Exclusion criteria of RRMS patients and HCs were as follows: individuals with a BMI more than 30.0 (kg/m^2^) or age lower than 20 years; patients with chronic conditions such as hypertension, hyperlipidemia, and diabetes mellitus; patients diagnosed with active infection such as bacteria, virus, and fungi; patients diagnosed with another autoimmune illness or neurological diseases.

**Table 1:** Demographic and clinical characteristics of RRMS patients and healthy controls.

Characteristics	RRMS	HCs	*P* value
Number	20	22	NA
Age (years), mean ± SD.	34.75 ± 7.59	33.36 ± 8.30	NS
Gender, male/female	8/12	8/14	NS
BMI (kg/m^2^), mean ± SD.	21.6 ± 2.6	22.3 ± 3.6	NS
MS duration (years), mean ± SD.	8.15 ± 5.0	NA	NA
Received treatment with immuno-modulatory drugs within 20 months	0	0	NS
Hypertension	0	0	NS
Hyperlipidemia	0	0	NS
Diabetes mellitus	0	0	NS
Active bacterial, fungal, or viral infections	0	0	NS
Autoimmune illnesses	0	0	NS
RRMS comorbidity with other neurological diseases	0	0	NS

RRMS, relapsing-remitting multiple sclerosis; HCs, healthy controls; BMI, body mass index; SD, standard deviation; NA, not available; NS, not significant.

Plasma samples were collected within 5 days of relapse onset and before initiation of DMT. Approximately 4.0 mL of EDTA-anticoagulant peripheral blood was collected from each subject before RRMS patients received DMT, then, all blood samples were centrifuged at 3,500× g and 4 °C for 10 minutes (min), to completely separate plasma and blood cells, the plasma was pipetted into a new 1.5 mL tube and frozen quickly in the liquid nitrogen. Immediately, all plasma samples were stored at -80 °C for follow-up analysis.

### 2.2 Untargeted metabolomics analysis

#### 2.2.1 Preparation of NIST serum standard curve calibration solutions

First, add 5, 10, 50, 100, 200, and 300 μL of NIST (National Institute of Standards and Technology) serum (standard reference material^®^ 1950) to centrifuge tubes, respectively, and supplement with ddH_2_O, and methanol (ThermoFisher Scientific, USA) to a certain ratio. A mixture of 11 deuterated and 13C-labeled internal standards (including d31-palmitic acid, d3-oleic acid, d8-arachidonic acid, d5-tryptophan, d3-leucine, d8-phenylalanine, d4-cholic acid, d4-glycocholic acid, 13C-lysophosphatidylcholine 16:0, and 13C-phosphatidylethanolamine 18:0/18:1) was added to each sample at a final concentration of 2 µg/mL for absolute quantification and quality control (QC). Vortex the six NIST serum samples thoroughly. Centrifuge samples at 13,500× g and 4 °C for 10 min, then transfer the supernatant of each sample to another 2 mL centrifuge tube. Next, concentrate samples to dryness under vacuum, redissolve in 150 μL of 80% methanol (pre-cooled to -20 °C), and centrifuge again at 13,500× g and 4 °C for 10 min to obtain the supernatant for liquid chromatography-tandem mass spectrometry (LC-MS/MS) analysis using the high-performance liquid chromatography (HPLC) Ultimate 3000 system and Q Exactive Focus mass spectrometer (ThermoFisher Scientific, USA).

#### 2.2.2 Extraction of plasma total metabolites

All plasma samples were thawed at 4 °C, and 100 μL of each sample was transferred to a new 2 mL centrifuge tube. Then, 100 uL of the mixed internal standard solution and 400 uL of methanol (pre-cooled at -20 °C) were added to each sample, which was vortexed for 60 seconds and centrifuged at 13,500× g and 4 °C for 10 min. Subsequently, 500 uL of the supernatant from each sample was transferred to another new 2 mL centrifuge tube. All samples were concentrated to complete dryness under vacuum, dissolved in 150 uL of 80% methanol, and centrifuged again at 13,500× g and 4 °C for 10 min. The supernatant was pipetted to a new centrifuge tube for LC-MS/MS analysis. QC samples were used to monitor any deviations in the analysis (52, 53).

#### 2.2.3 Chromatographic separation assay

A Thermo Ultimate 3000 system coupled with an ACQUITY UPLC^®^ HSS T3 column (2.1×150 mm×1.8 μm) (Waters Corporation Ltd., Milford, USA) was used to perform chromatographic separation assay. The column oven temperature was maintained at 40 °C. Mobile phases were prepared using 0.1% formic acid (TCI, Inc., Tokyo, Japan) in ddH_2_O (Millipore, Inc.) and acetonitrile (ThermoFisher Scientific, USA), or 5.0 mM ammonium formate (Sigma, Inc.) in ddH_2_O and acetonitrile. Gradient elution was performed at a flow rate of 0.25 mL/min. After equilibration, 2.0 μL of each sample was injected. Blank samples (methanol only) were injected every 10 samples to monitor system carryover and background contamination. Any features detected in blanks with intensity > 30% of median sample intensity were flagged as potential contaminants and removed from the dataset.

#### 2.2.4 Mass spectrometry assay

Mass spectrometry assay was performed according to well-established protocols (54). A Thermo Q Exactive mass spectrometer (ThermoFisher Scientific, USA) used was to detect metabolites in both positive and negative ESI modes. Simultaneous MS1 and MS/MS (full MS mode, data-dependent acquisition, DDA) were employed. Parameters were set as follows: sheath gas pressure at 30 arbitrary (arb) units, auxiliary gas pressure at 10 arb units; spray voltage at 3.50 kV for ESI (+) and -2.50 kV for ESI (-); capillary temperature at 325 °C; MS1 scan range from mass/charge (*m*/*z*) 81-1,000; MS1 resolution at 70,000 FWHM; three scans per cycle; MS/MS resolution at 17,500 FWHM; normalized collision energy at 30 eV; dynamic exclusion time was set to automatic.

For data acquisition, compounds were separated via column chromatography and data was acquired through mass spectrometry. Each scan generated a chromatogram with ion intensity on the y-axis and time on the x-axis. The most intense ions were continuously tracked to obtain the base peak chromatogram (BPC).

### 2.3 Bioinformatic analysis

#### 2.3.1 Raw data preprocessing and QC

Data Processing. The raw data was converted into mzXML format files using the MSConvert tool from ProteoWizard (v3.0.8789) (55). Then, the XCMS package (v.3.12.0) (56) in R was employed for peak detection, filtering, and alignment. Subsequent, a data matrix file was obtained, containing information such as *m*/*z* ratio, retention time, and relative peak area ratio.

Data QC. First, systematic errors were eliminated using support vector regression correction based on QC samples. Then, data filtering was conducted to remove low-quality data. Ions with a relative standard deviation (RSD) lower than 30% in the QC samples were retained for downstream analysis, otherwise, they are filtered out.

#### 2.3.2 Metabolite identification

Metabolite identification followed the Lipidomics Standards Initiative (LSI) reporting framework. For fatty acids, Level 1 identification was achieved by matching retention time (± 0.1 min), accurate mass (± 5 ppm), and MS/MS fragmentation patterns to authentic standards. For complex lipids, Level 2 identification was assigned based on accurate mass (± 5 ppm) and MS/MS spectral library matching (cosine similarity > 0.8) to databases including MoNA and MassBank. MS/MS data were acquired using data-dependent acquisition with collision energies of 20, 40, and 60 eV. Only metabolites with Level 1 or Level 2 confidence were included in downstream differential abundance analysis. Metabolite identification involved confirming molecular weights within a 15 ppm error margin, followed by matching against multiple databases: HMDB (http://www.hmdb.ca) (57), mzCloud (http://www.mzcloud.org) (58), MassBank (http://massbank.jp) (59), Lipid Maps (http://www.lipidmaps.org) (60), MoNA (https://mona.fiehnlab.ucdavis.edu), and Metlin (https://metlin.scripps.edu). Besides, a custom database, constructed by BioNovoGene Co., Ltd. (Suzhou, China) using MS/MS fragment data, was also employed. Identified metabolites were classified using Metabolon databases.

#### 2.3.3 Missing value imputation

For missing dataset values, the k-nearest neighbors (k-NN) imputation method was employed (61). k value was set to 10 following the recommendation of Gromski et al. (2014) for metabolomics data (62). This technique identifies each metabolite’s k-nearest neighbors and fills missing data with their average value. Subsequent analyses used the data with missing value imputation.

#### 2.3.4 Data transformation

To guarantee the robustness of data analysis, Log_2_ transformation was applied to raw metabolite data. Specifically, the data was transformed using the formula Log_2_(raw value + 1). This method stabilizes variance, mitigates extreme value effects, and retains information from low-metabolite samples. Subsequent analyses utilized the transformed data.

#### 2.3.5 Batch effect correction

To detect batch effects, we employed a multi-pronged approach: (1) Relative Log Expression (RLE) plots were generated to visualize systematic shifts in metabolite distributions across batches; (2) PCA score plots were color-coded by batch ID to visually inspect for batch-driven clustering; (3) the correlation between principal components and batch number was quantified; and (4) QC sample trends were monitored across the run order. Following confirmation of batch effects via these methods, the ComBat method from the SVA package (v.3.42.0) (63) was applied to adjust for batch variation using batch ID as a known covariate.

#### 2.3.6 Confounder correction

To simultaneously account for potential confounding effects of age and sex, we employed a multiple linear regression model for each metabolite: Metabolite_abundance ~ Sex + Age + ε, where Sex was coded as a binary variable (0 = female, 1 = male) and Age was continuous (in years). The residuals from this model (ε) represent metabolite abundances after simultaneous adjustment for both confounders. These residuals were used for all downstream univariate (t-tests, correlations) and multivariate (PCA, OPLS-DA) analyses. This approach ensures that the effects of age and sex are estimated simultaneously, avoiding order-dependency and providing more stable confounder correction.

#### 2.3.7 Data normalization

Data normalization in SIMCA-P software (v.14.1, Umetrics, Umea, Sweden) was configured as follows: (1) Distance to model: normalized by standard deviation units and weighted by modeling power; (2) Coefficients: scaled and centered; (3) Model fit parameters: R2X (cumulative variation in metabolite data explained by the model) and R2Y (cumulative variation in class membership explained by the model) were calculated; (4) Residuals were standardized for diagnostic purposes.

#### 2.3.8 Multivariate statistical analysis

Before conducting multivariate analysis, metabolomics data were subject to auto-scaling, which involves mean-centering and unit-variance scaling. This scaling method equalizes each metabolite’s contribution to the analysis, irrespective of its initial concentration. Specifically, each metabolite’s data underwent mean-centering (subtracting the mean value across all samples) and scaling to unit variance (dividing by the standard deviation across all samples). Scaling was done to transform metabolome data into suitable weights and to achieve more trustworthy and understandable results prior to multivariate analysis. In this study, the Ropls package (v.1.22.0) (64) in R was used to conduct multivariate statistical analysis. Specifically, the unsupervised analysis such as PCA, and the supervised analysis involved the use of partial least squares discriminant analysis (PLS-DA) and orthogonal PLS-DA (OPLS-DA) (65) to analyze all samples. Pareto scaling (mean-centering + square root of standard deviation) was used for OPLS-DA to preserve biological information while reducing noise. PLS-DA and OPLS-DA models were validated using 7-fold cross-validation repeated 10 times. The final OPLS-DA model achieved R²X = 0.63, R²Y = 0.85, and Q² = 0.72. Permutation testing (200 permutations) confirmed model validity with pR2Y < 0.001 and pQ2 = 0.002. The Q² intercept of -0.23 indicates that the model performs significantly better than random chance.

#### 2.3.9 Identification of differentially abundant metabolites

Differentially abundant metabolites (DAMs) were identified using a two-tiered approach: (1) Multivariate significance: Variable Importance in Projection (VIP) score > 1.00 derived from the OPLS-DA model; and (2) Univariate significance: Two-tailed Welch’s t-test with *P* values adjusted for false discovery rate (FDR) using the Benjamini-Hochberg (BH) procedure, with significance threshold set at FDR-adjusted *P* (*P_adj_*) value < 0.05. Only metabolites meeting both criteria were designated as DAMs.

For differential analysis, Z-scores were computed based on metabolite levels relative to the control group’s mean and standard deviation. This standardizes the data for comparison and measures the relative metabolite content. Pearson correlations between metabolites and cytokines were calculated using the rcorr() function from the Hmisc package (v4.7-0) in R, which provides asymptotic *P* values. To account for multiple testing across 224 correlations, *P* values were adjusted using the BH FDR procedure, with significance threshold set at *P_adj_* < 0.05. Only correlations meeting both the raw *P* < 0.05 and *P_adj_* < 0.05 criteria are reported as significant. The pROC package (v.1.18.0) (66) in R was used for receiver operator characteristic (ROC) curve analysis, which evaluated potential biomarkers via the area under the curve (AUC). Leave-One-Out Cross-Validation (LOOCV) was used to maximize training data while providing rigorous internal validation. MetPA in MetaboAnalyst 4.0 (www.metaboanalyst.ca) analyzed metabolic pathways using hypergeometric tests. ggplot2 package (v.3.3.4) in R was used to draw violin plot. Kyoto Encyclopedia of Genes and Genomes (KEGG) pathway enrichment analysis was performed using clusterProfiler (v.4.2.2) in R (67–69).

#### 2.3.10 Hierarchical clustering analysis

Hierarchical clustering analysis (HCA) was performed using Euclidean distance to calculate pairwise distances between samples, followed by average linkage clustering. The distance matrix and clustering were computed using the pheatmap package (v1.0.12) (70) in R, with metabolite data auto-scaled prior to distance calculation. The resulting heatmap visualized both metabolite and sample clustering patterns.

### 2.4 Measurement of cytokine and chemokine levels

The magnetic beads-based immunoassay kit (Bio-Rad, CA, USA), used with the Bio-Plex 200 system, measured the concentrations of these cytokines and chemokines: tumor necrosis factor-α (TNF-α), interleukin 17A (IL17A), interleukin 7 (IL7), interleukin 9 (IL9), interleukin 12A (IL12A), interleukin 13 (IL13), interleukin 1 receptor antagonist (IL1RA), C-C motif chemokine ligand 2 (CCL2), C-C motif chemokine ligand 3 (CCL3), C-C motif chemokine ligand 4 (CCL4), C-C motif chemokine ligand 5 (CCL5), C-C motif chemokine ligand 11 (CCL11), platelet derived growth factor subunit B (PDGFB), C-X-C motif chemokine ligand 8 (CXCL8), C-X-C motif chemokine ligand 10 (CXCL10), and Interferon γ (IFNG). Cytokine/chemokine concentrations were Log_2_-transformed to achieve approximate normality prior to statistical testing and visualization.

### 2.5 Correlation analysis of fatty acids and immuno-inflammatory factors

Mantel tests and Pearson correlation analysis were used to assess correlations between fatty acids (e.g., FA 20:4 and FA 16:0) and immune-inflammatory factors (e.g., TNF-α and IL17A). Additionally, linear regression models were used to determine the strength and statistical significance of correlations between specific fatty acids (e.g., FA 20:4) and cytokines/chemokines (e.g., IL17A and CCL2).

### 2.6 Statistical analysis

Between-group comparisons of metabolite and cytokine levels were performed using a two-tiered statistical approach to ensure both parametric validity and control of false discoveries across the high-dimensional dataset. All analyses were conducted on confounder-corrected data (residuals after simultaneous adjustment for age and sex) using R statistical software (v4.2.1).

Prior to statistical testing, the distribution of each metabolite and cytokine was assessed for normality using the Shapiro-Wilk test on log_2_-transformed, imputation-adjusted, and confounder-corrected data. Normality assessment results demonstrated data normal distribution (Shapiro-Wilk *p* > 0.05) and homogeneity of variance (Levene’s test *p* > 0.05), thus differences between the RRMS and HC groups were evaluated using independent two-sample t-tests. GraphPad Prism (v.9.3) was used for figure assembly.

Given the high-dimensional nature of metabolomics data, all *P* values from univariate comparisons were corrected for multiple testing using the BH FDR procedure via the p.adjust() function in R. The FDR threshold was set at *P_adj_* value < 0.05. This correction was applied globally across all metabolite-level tests and separately across all cytokine comparisons to control the expected proportion of false positives while maintaining statistical power appropriate for exploratory biomarker discovery.

## 3 Results

### 3.1 Identification of differentially abundant metabolites in RRMS patients

**Figure 1:**
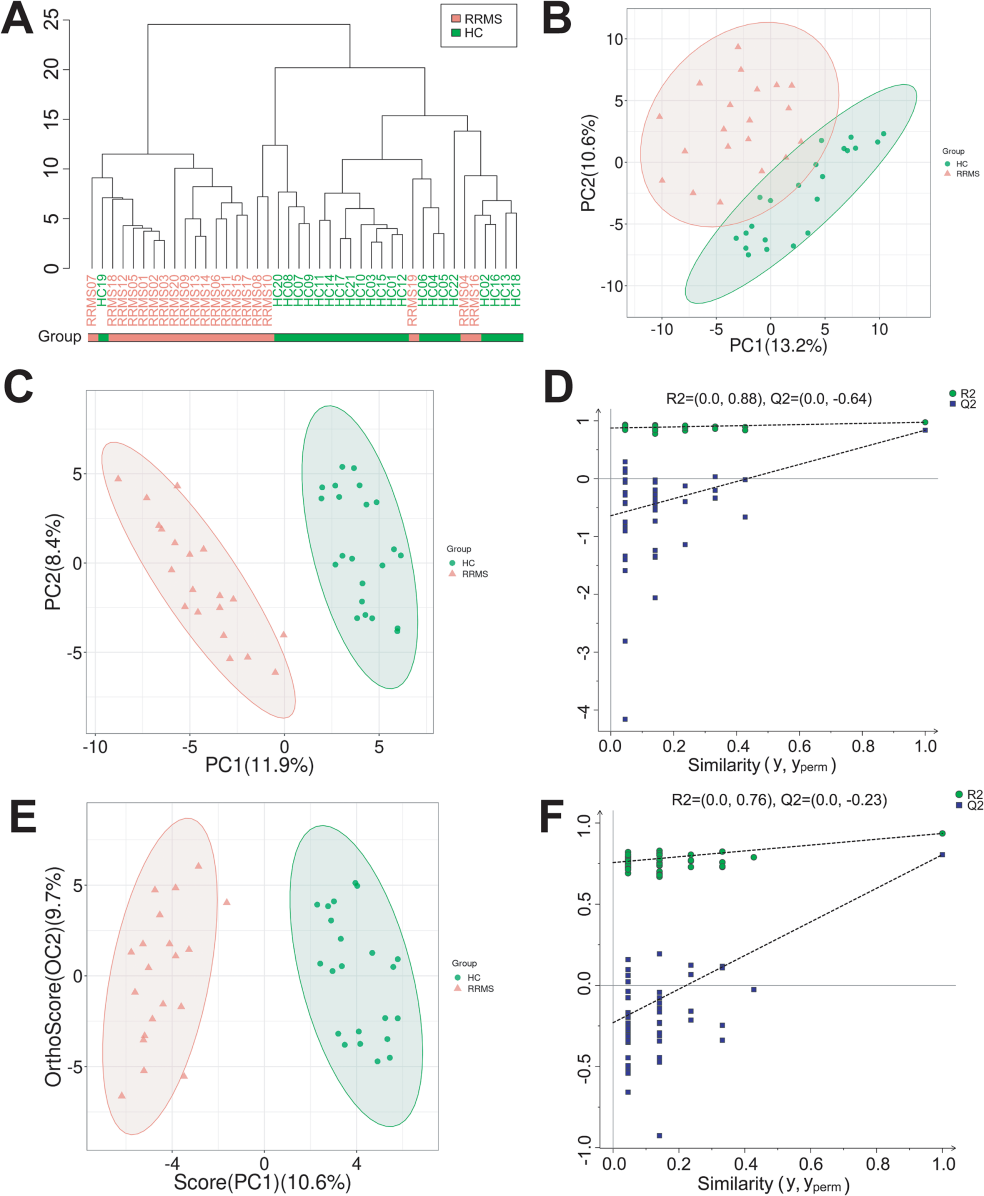
PCA, PLS-DA, and OPLS-DA models for separating RRMS and HC samples. **(A)** Hierarchical cluster analysis of RRMS and HC group samples. **(B)** Unsupervised PCA analysis showed RRMS were properly separated from HC samples. Pre =8, R2X = 0.531. **(C, D)** Supervised PLS-DA analysis represented RRMS were completely separated from HC group. R2X = 0.270, R2Y = 0.975, Q2 = 0.838. **(E, F)** Supervised OPLS-DA analysis demonstrated RRMS were clearly separated from HC group. R2X = 0.203, R2Y = 0.936, Q2 = 0.805.

To investigate the metabolism changes of fatty acids, particularly MUFA and PUFA, in Chinese RRMS patients, we used LC-MS/MS-based untargeted metabolomics approach to analyze plasma samples from 20 RRMS patients and 22 age- and sex-matched healthy controls (HCs) subjects. The characteristics and demographic information of RRMS patients and HCs was presented in **Table 1**. Hierarchical clustering analysis revealed that samples primarily grouped by disease status, with RRMS and HC forming distinct clusters (**Figure 1A**). Unsupervised PCA analysis revealed that RRMS and HC groups showed partial separation along PC1 (13.2%), with considerable overlap (**Figure 1B**). While group centroids differed obviously, the overlap suggests substantial inter-individual variability and that PCA alone was insufficient for classification, justifying the need for supervised methods. Supervised analysis PLS-DA (**Figures 1C, D**) and OPLS-DA (**Figures 1E, F**) showed complete separation between the RRMS and HC samples, indicating significant metabolic changes in RRMS patients. The OPLS-DA model indicated good fit and predictive ability. However, the high R² intercept in permutation testing suggests potential overfitting, which we address through cross-validation and acknowledge as a limitation requiring external validation.

**Figure 2:**
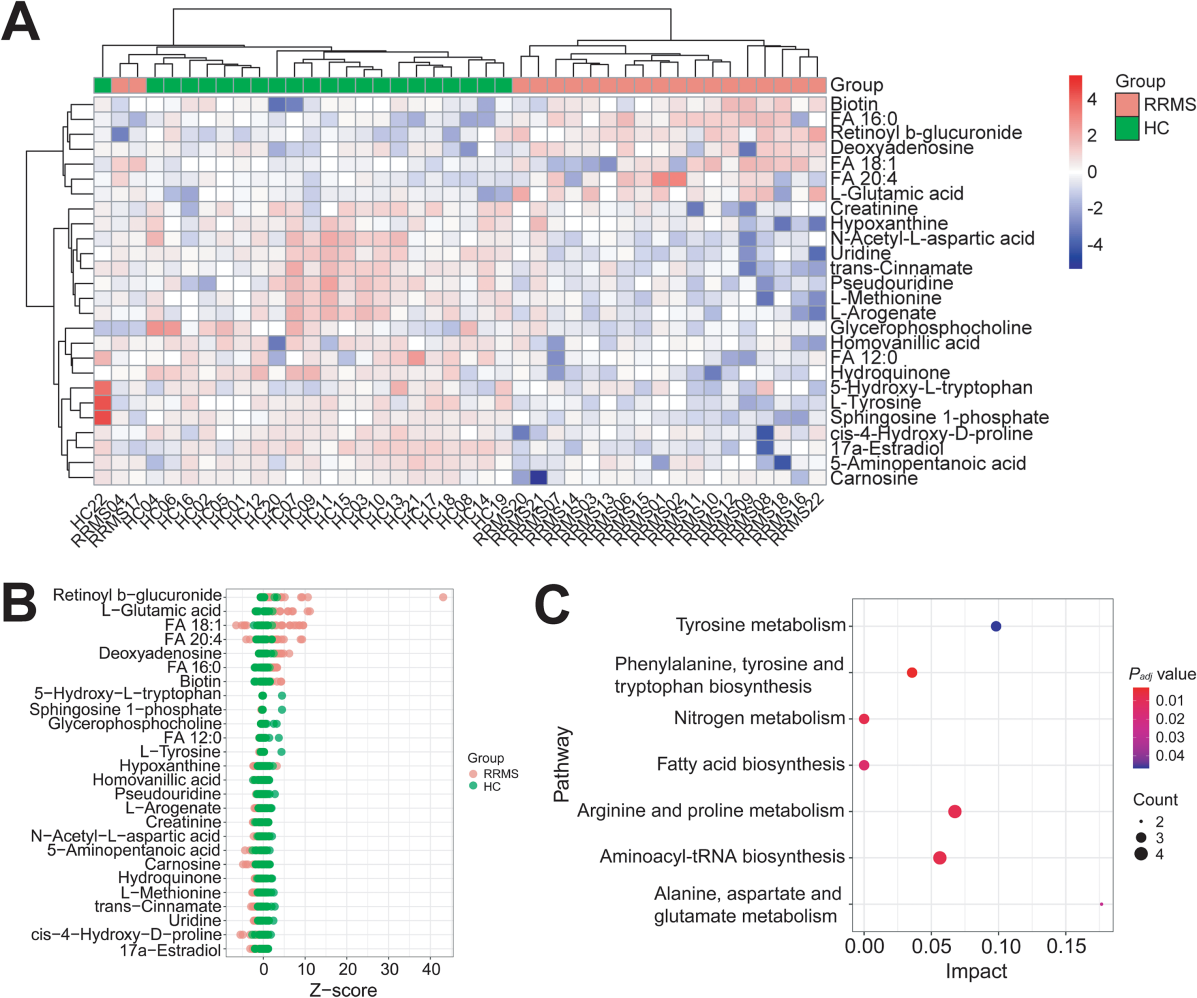
Twenty-six differentially abundant metabolites were identified in RRMS compared to HCs. **(A)** Heatmap showed twenty-six differentially abundant metabolites (DAMs) (VIP > 1.00, *P_adj_* value < 0.05) in RRMS samples relative to HCs. The relative levels (Log_2_ transformed) of all DAMs were determined using Pheatmap package (v.1.0.12) in R. **(B)** Z-score plot represented seven up-regulated and nineteen down-regulated DAMs in RRMS relative to HCs. **(C)** Bubble diagram showed the twenty-six DAMs were significantly enriched in multiple pathways, such as fatty acid biosynthesis, tyrosine metabolism, and so on. Pathway enrichment analysis showing only significantly enriched pathways (*P_adj_* < 0.05). Impact score combines enrichment significance and pathway coverage calculated by MetaboAnalyst.

To identify differentially abundant metabolites (DAMs) significantly related to RRMS, we set VIP > 1.0 and *P_adj_* < 0.05 as criteria to screen DAMs. Compared to HCs, a total of 26 DAMs were identified in RRMS samples, including 7 upregulated DAMs such as FA 20:4 (VIP = 1.275, Log_2_FC = 0.031, *P_adj_* < 0.01), FA 18:1 (VIP = 1.195, Log_2_FC = 0.283, *P_adj_* < 0.05), FA 16:0 (VIP = 1.617, Log_2_FC = 0.193, *P_adj_* < 0.001), L-Glutamic acid (VIP = 1.872, Log_2_FC = 1.188, *P_adj_* < 0.001), Biotin (VIP = 1.188, Log_2_FC = 0.481, *P_adj_* < 0.05), Retinoyl b-glucuronide (VIP = 1.607, Log_2_FC = 3.045, *P_adj_* < 0.05), and Deoxyadenosine (VIP = 1.375, Log_2_FC = 1.273, *P_adj_* < 0.05), and 19 downregulated DAMs such as FA 12:0 (VIP = 1.766, Log_2_FC = -1.270, *P_adj_* < 0.01), 5-Aminopentanoic acid (VIP = 1.937, Log_2_FC = -0.290, *P_adj_* < 0.01), Homovanillic acid (VIP = 1.957, Log_2_FC = -0.586, *P_adj_* < 0.01), Uridine (VIP = 2.085, Log_2_FC = -1.178, *P_adj_* < 0.001), L-Tyrosine (VIP = 1.852, Log_2_FC = -0.829, *P_adj_* < 0.01), L-Methionine (VIP = 1.908, Log_2_FC = -0.763, *P_adj_* < 0.01), L-Arogenate (VIP = 1.719, Log_2_FC = -0.715, *P_adj_* < 0.01), N-Acetyl-L-Aspartic acid (VIP = 1.771, Log_2_FC = -0.544, *P_adj_* < 0.01), trans-Cinnamate (VIP = 1.869, Log_2_FC = -0.598, *P_adj_* < 0.01), and so on (**Figures 2A, B**; **Supplementary Figure 1**). KEGG pathway enrichment analysis showed these DAMs were significantly enriched in several pathways, including fatty acid metabolism, tyrosine metabolism, phenylalanine, tyrosine and tryptophan biosynthesis, nitrogen metabolism, arginine and proline metabolism, aminoacyl-tRNA biosynthesis, and alanine, aspartate and glutamate metabolism (**Figure 2C**).

### 3.2 Significant alterations in LCFAs metabolism in relapsed RRMS patients

**Figure 3:**
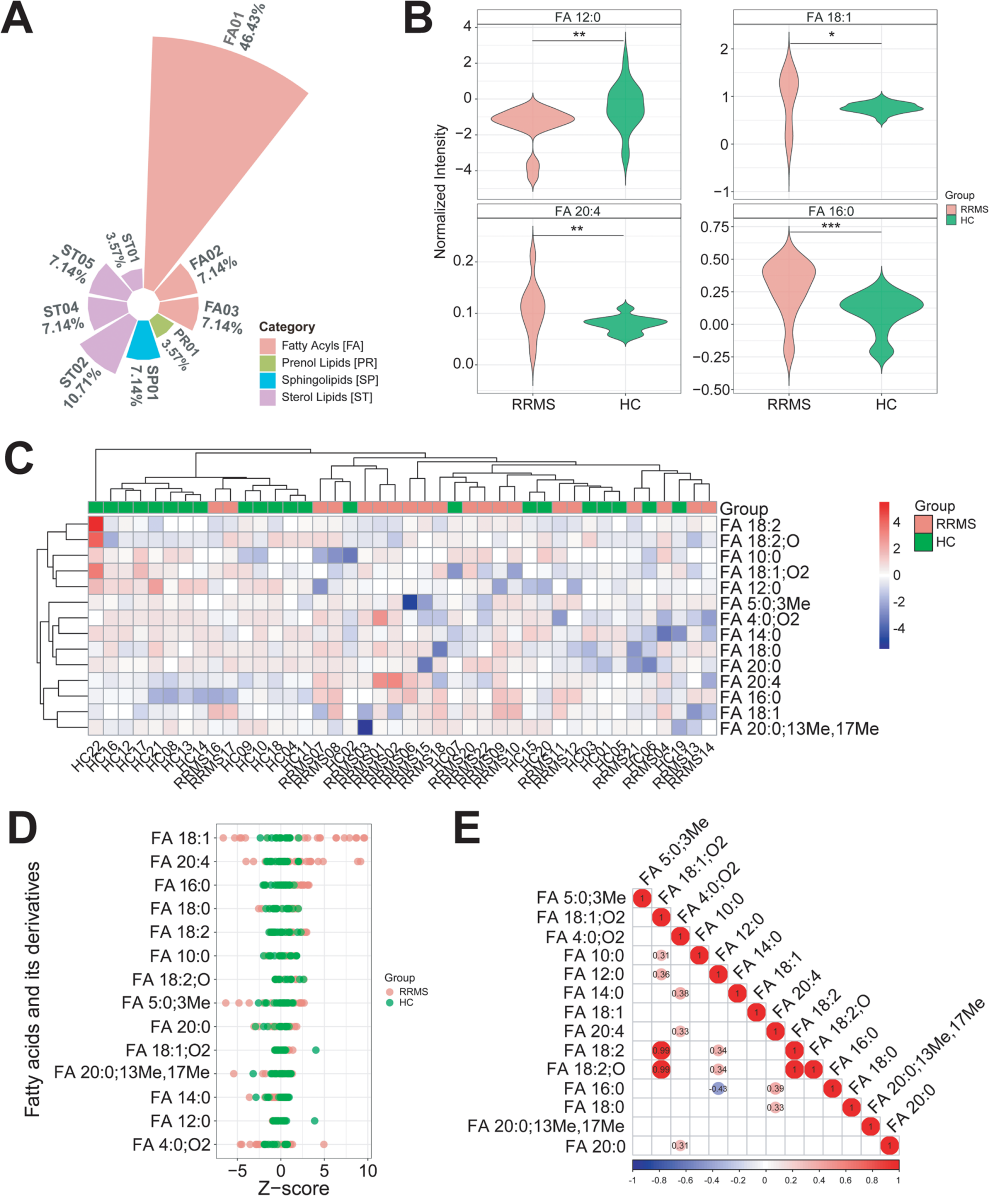
Dysregulated fatty acid metabolism in the peripheral system of relapsed RRMS patients. **(A)** Radar chart showed that the vast majority of all fatty acid metabolites belong to fatty acyls (FA), followed by sterol lipids (ST), sphingolipids (SP), and prenol lipids (PR). FA01: Fatty acids and conjugates, FA02: Fatty amides, FA03: Fatty amides (including N-acyl amines and N-acyl ethanolamines), PR01: Isoprenoids, SP01: Sphingoid bases, ST01: Sterols, ST02: Steryl esters, ST03: Steryl glycosides, ST04: Bile acids and derivatives, ST05: Acyl steryl glycosides. **(B)** Violin plot represented the circulating levels of FA 12:0, FA 18:1, FA 20:4, and FA 16:0 was significantly reduced and increased in RRMS compared to HCs, respectively. The normalized intensity values were Log_2_ transformed. RRMS and HC samples were compared using independent t-tests. *P* values were FDR-corrected using BH method. *: *P_adj_* < 0.05, **: *P_adj_* < 0.01, ***: *P_adj_* < 0.001. **(C)** Heatmap showed representative LCFA, MCFA, SCFA and their derivates identified in RRMS and HC samples. The relative levels (Log_2_ transformed) of all fatty acids were determined using Pheatmap package (v.1.0.12) in R. **(D)** Z-score plot represented the changes in plasma levels of representative LCFA, MCFA, SCFA and their derivates in RRMS and HCs. **(E)** Pearson’s correlation heatmap showed the correlation coefficient between any two fatty acid metabolites. Positive and negative correlation was represented by red and blue, respectively.

To further investigate the characteristics of metabolic profile of fatty acids, particularly LCFAs, in RRMS patients, we classified the fatty acids and lipid metabolites identified in RRMS samples. Results showed the main categories were fatty acyls (60.71%), followed by sterol lipids (28.56%), sphingolipids (7.14%), and prenol lipids (3.57%) (**Figure 3A**, **Supplementary Table 1**). Besides the significantly downregulated medium-chain saturated fatty acid FA 12:0 (Log_2_FC = -1.270, *P_adj_* < 0.01), and the significantly upregulated MUFA FA 18:1 (Log_2_FC = 0.283, *P_adj_* < 0.05), PUFA FA 20:4 (Log_2_FC = 0.031, *P_adj_* < 0.01), and long-chain saturated fatty acid FA 16:0 (Log_2_FC = 0.193, *P_adj_* < 0.001) (**Figure 3B**), we also identified other LCFAs such as myristic acid (FA 14:0), linoleic acid (FA 18:2), stearic acid (FA 18:0), bovinic acid (FA 20:0;13Me,17Me), arachidic acid (FA 20:0), SCFA isovaleric acid (FA 5:0;3Me), medium-chain fatty acid (MCFA) capric acid (FA 10:0), and other fatty acid derivatives (**Figures 3C, D**; **Supplementary Table 2**). Pearson correlation analysis showed significant correlations between different fatty acids. For instance, FA 18:2 and α-dimorphecolic acid (FA 18:2;O) levels were significantly positively correlated (r = 0.99, *P_adj_* < 0.001), as were FA 18:2 and 12,13-DiHOME (FA 18:1;O2) (r = 0.99, *P_adj_* < 0.001), and FA 18:2;O and FA 18:1;O2 (r = 0.99, *P_adj_* < 0.001). FA 18:2 is indeed the precursor for FA 18:2;O (namely 9-HODE, a hydroxy-octadecadienoic acid) via enzymatic oxidation (71, 72), while FA 18:1;O2 is a diol metabolite of FA 18:2 generated via cytochrome P450 epoxygenase and soluble epoxide hydrolase pathways (72), functioning as a bioactive oxylipin that modulates metabolic and inflammatory processes (73). The near-perfect correlation (r = 0.99) was biochemically plausible and reflects their precursor-product relationship. In contrast, FA 16:0 and FA 12:0 levels showed significant negative correlation (r = -0.43, *P_adj_* < 0.01) (**Figure 3E**).

### 3.3 TNF-α and IL17A-mediated inflammatory responses was enhanced in RRMS patients

**Figure 4:**
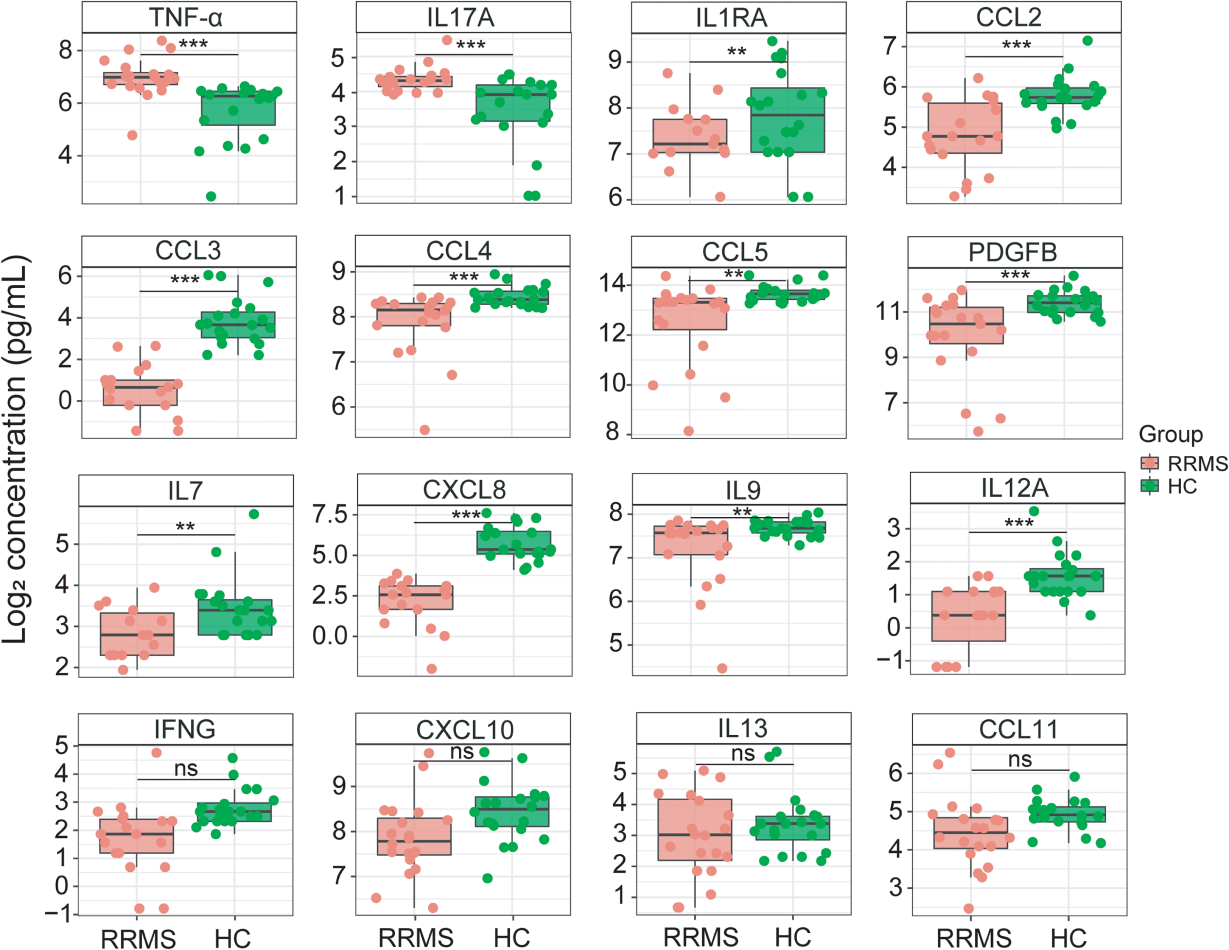
Significant changes in the plasma levels of pro-inflammatory cytokines and chemokines in RRMS patients. The quantitative concentrations (pg/mL) of TNF-α, IL17A, IL1RA, CCL2, CCL3, PDGFB, CCL4, CCL5, IL7, CXCL8, IL9, IL12A, IFNG, CXCL10, IL13, and CCL11 in plasma samples of RRMS patients and HCs. Log_2_ transformation was performed on the concentrations of all cytokine/chemokine. The y-axis represents Log_2_-transformed values. Samples between the two groups were compared using independent t-tests. *P* values were FDR-corrected using BH method. **: *P_adj_* < 0.01, ***: *P_adj_* < 0.001. ns: not significant.

**Figure 5:**
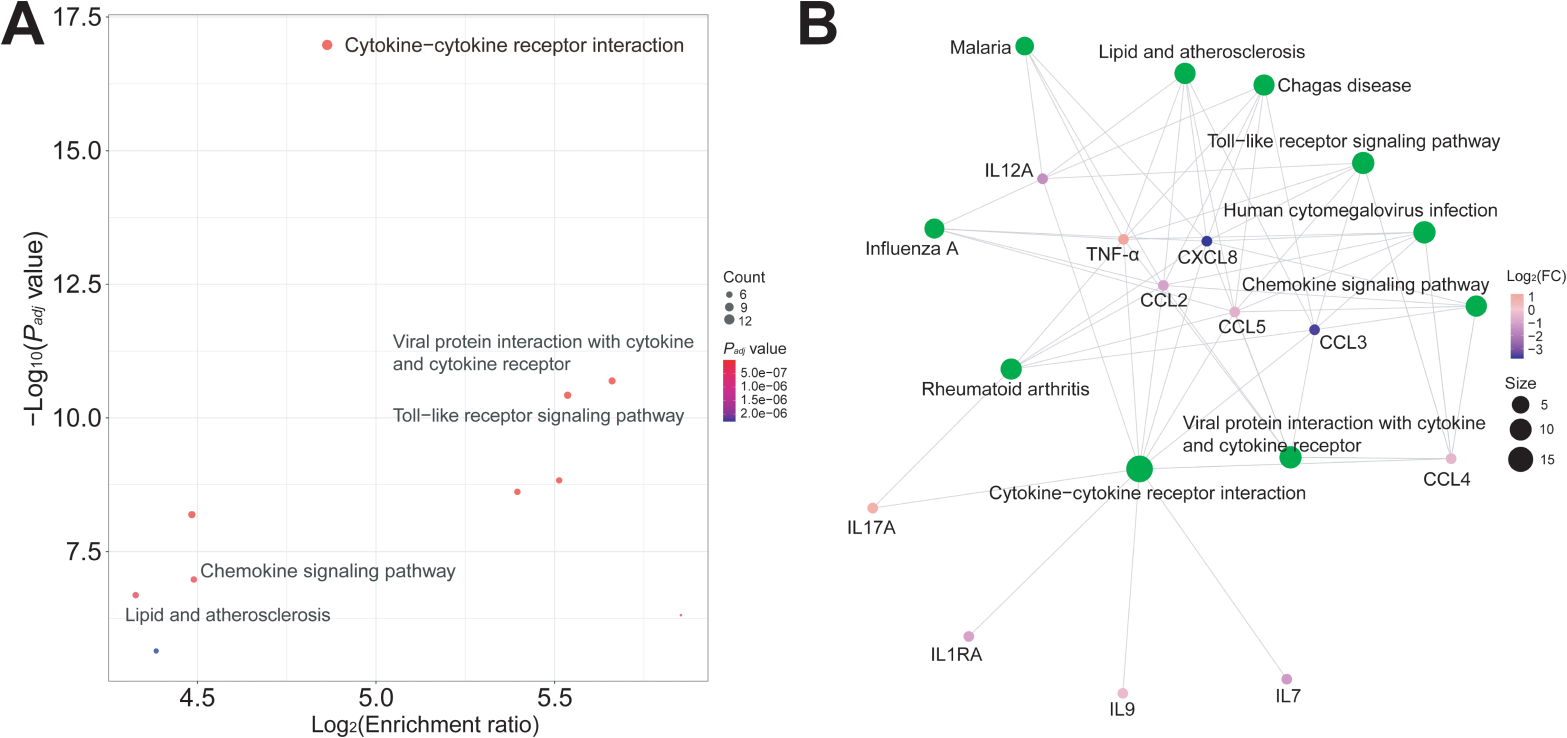
Functional and pathway enrichment analysis of significantly expressed immune-inflammatory factors in RRMS patients. **(A)** Bubble plot showed differentially expressed cytokines/chemokines were significantly enriched in multiple pathways, including cytokine-cytokine receptor interaction, viral protein interaction with cytokine and cytokine receptor, chemokine signaling pathway, and so on. *P* values are FDR-adjusted via BH correction. Only pathways with *Padj* < 0.05 are displayed. **(B)** Network diagram represented the interaction between differentially expressed cytokines/chemokines and their participating pathways. KEGG pathway enrichment analysis was performed using clusterProfiler (v.4.2.2) in R.

To investigate the changes of peripheral immune-inflammatory responses in RRMS patients, we detected pro-inflammatory cytokines (e.g., TNF-α and IL17A) and pro-inflammatory chemokines (e.g., CCL2 and CCL3) in plasma samples of RRMS patients and HCs. Results showed that compared to HCs, the circulating levels of TNF-α (Log_2_FC = 1.159, Cohen’s d = -1.415, *P_adj_* < 0.001) and IL17A (Log_2_FC = 0.750, Cohen’s d = -1.268, *P_adj_* < 0.001) were significantly elevated in RRMS samples, while IL1RA (Log_2_FC = -1.012, Cohen’s d = 0.894, *P_adj_* < 0.01), CCL2 (Log_2_FC = -1.002, Cohen’s d = 1.325, *P_adj_* < 0.001), CCL3 (Log_2_FC = -3.506, Cohen’s d = 1.326, *P_adj_* < 0.001), PDGFB (Log_2_FC = -0.951, Cohen’s d = 1.212, *P_adj_* < 0.001), CCL4 (Log_2_FC = -0.466, Cohen’s d = 1.368, *P_adj_* < 0.001), CCL5 (Log_2_FC = -0.650, Cohen’s d = 1.076, *P_adj_* < 0.01), IL7 (Log_2_FC = -1.224, Cohen’s d = 0.898, *P_adj_* < 0.01), CXCL8 (Log_2_FC = -3.743, Cohen’s d = 1.709, *P_adj_* < 0.001), IL9 (Log_2_FC = -0.319, Cohen’s d = 0.888, *P_adj_* < 0.01), and IL12A (Log_2_FC = -1.529, Cohen’s d = 1.284, *P_adj_* < 0.001) levels were significantly reduced (**Figure 4**; **Supplementary Table 3**). The majority of cytokines/chemokines showed statistical significance (*P_adj_* < 0.05), and their effect sizes were predominantly large-to-very large (Cohen’s d = 0.888-1.709). Among them, TNF-α (d = -1.415), IL17A (d = -1.268), CXCL8 (d = 1.709), CCL4 (d = 1.368), CCL3 (d = 1.326), and CCL2 (d = 1.325) demonstrated very large effects, suggesting these pro-inflammatory factors may have greater clinical relevance as biomarkers of MS relapse activity. KEGG pathway enrichment analysis showed these differentially expressed cytokines and chemokines were mainly enriched in cytokine-cytokine receptor interaction, viral protein interaction with cytokine and cytokine receptor, chemokine signaling pathway, Toll-like receptor signaling pathway, and lipid and atherosclerosis pathways (**Figures 5A, B**).

### 3.4 Significant link between LCFA and immuno-inflammatory responses in RRMS patients

**Figure 6:**
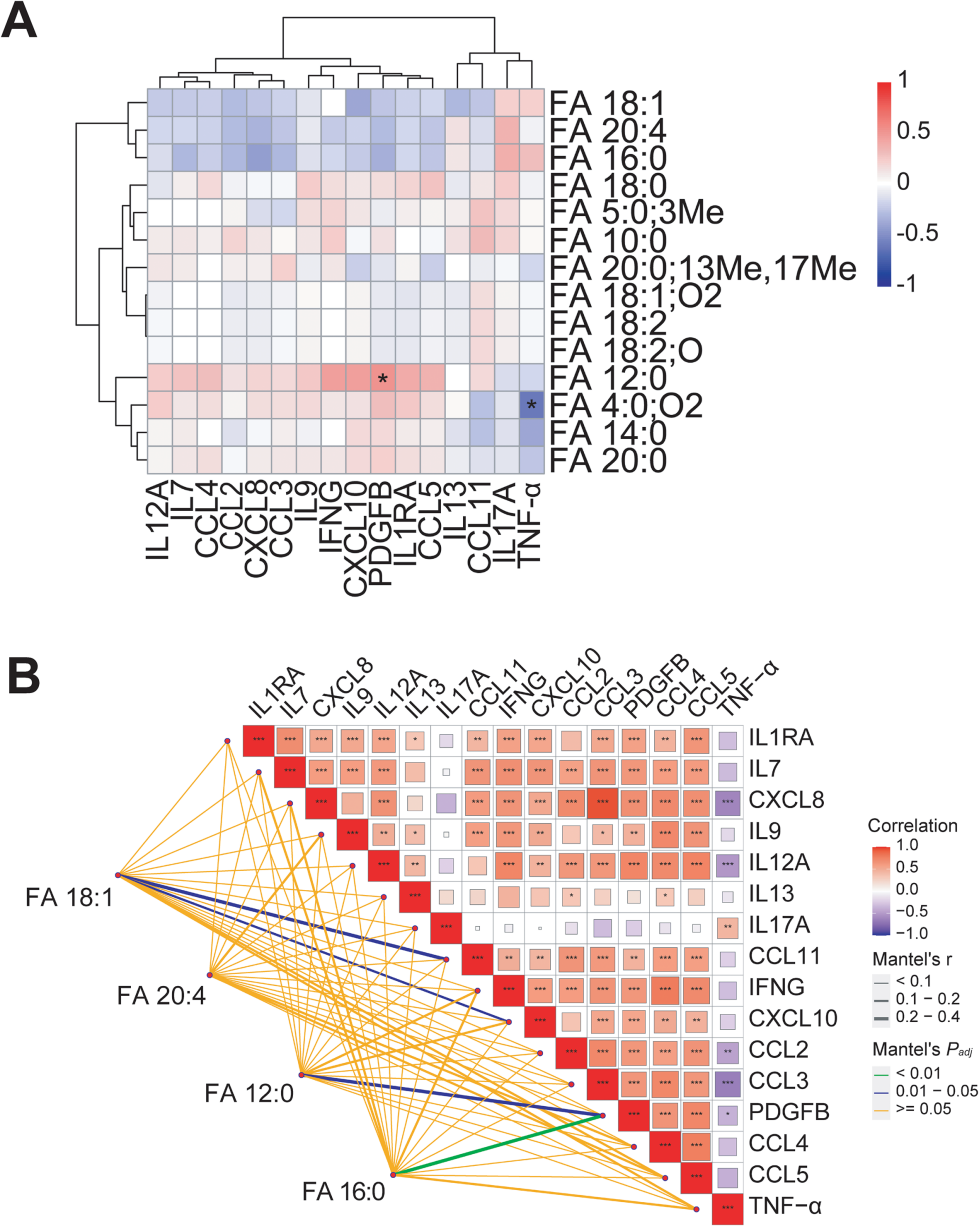
Modest correlations between LCFA metabolites and immune-inflammation regulatory factors during MS relapse period. **(A)** Pearson’s correlation analysis showed the correlation between fourteen fatty acid (such as LCFA FA 18:2 and FA 18:0) metabolites/derivatives and sixteen cytokines/chemokines. Asterisks indicate correlations surviving FDR correction (*P_adj_* < 0.05) across 224 tests. Color intensity reflects correlation coefficient magnitude; only *P_adj_* < 0.05 correlations are highlighted with asterisks (*). **(B)** Mantel tests analysis demonstrated that LCFAs such as FA 18:1 and FA 16:0 significantly correlated to CCL11, CXCL10, and PDGFB, respectively. **: Padj < 0.01, ***: Padj < 0.001.

To further explore the interplay between peripheral metabolism dysregulation of fatty acid in particular LCFAs and immune-inflammatory responses, and their impact on RRMS progression, we analyzed the interactive networks and correlation between fatty acids and their derivatives, including LCFAs such as FA 16:0, FA 18:1, FA 20:4, FA 14:0, FA 18:2, FA 18:0, FA 20:0;13Me,17Me, and FA 20:0, MCFAs such as FA 12:0, and FA 10:0, SCFAs like FA 5:0;3Me, and fatty acid derivatives like FA 18:2;O, and pro-inflammatory cytokines (e.g., TNF-α and IL17A) and pro-inflammatory chemokines (e.g., CCL2 and CCL3). Pearson correlation analysis showed a significant positive correlation between reduced FA 12:0 levels and decreased PDGFB concentrations (r = 0.522, *P_adj_* < 0.05), and a significant negative correlation between lower succinic acid (FA 4:0;O2) levels and higher TNF-α concentrations (r = -0.613, *P_adj_* < 0.05) (**Figure 6A**), despite no significant difference in FA 4:0;O2 levels between RRMS patients and HCs. Mantel correlation analysis showed FA 18:1 levels were modestly but significantly correlated with CCL11 (r = 0.300, *P_adj_* < 0.05) and CXCL10 (r = 0.197, *P_adj_* < 0.05) concentrations (**Figure 6B**), even though the changes in CCL11 and CXCL10 levels in RRMS patients were not significant (**Figure 4**). FA 16:0 levels showed a modest and significant correlation with PDGFB concentration changes (r = 0.202, *P_adj_* < 0.01), and FA 12:0 levels were modestly and significantly correlated with PDGFB concentration changes (r = 0.200, *P_adj_* < 0.05) (**Figure 6B**). The weak Mantel’s r values (0.10-0.40) indicate modest overall pattern similarity, supporting the concept of systemic metabolic-immune crosstalk while acknowledging that individual lipid-cytokine relationships explain limited variance. To further exclude the influence of group differences on fatty acid-cytokine relationships, we performed correlation analyses within the RRMS and HC groups separately. The results revealed 15 significant correlations within the RRMS group (e.g., FA 4:0;O2 with TNF-α, and FA 18:2 with CXCL8) (*P* < 0.05, **Supplementary Figure 2A**). In contrast, only 5 significant correlations were observed within the HC group (*P* < 0.05, **Supplementary Figure 2B**), indicating that most fatty acid-cytokine correlations were not driven by group differences. These results indicated a significant link between altered LCFAs and MCFAs levels and immune-inflammatory responses, suggesting LCFAs dysregulation may modulate peripheral immune responses.

**Figure 7:**
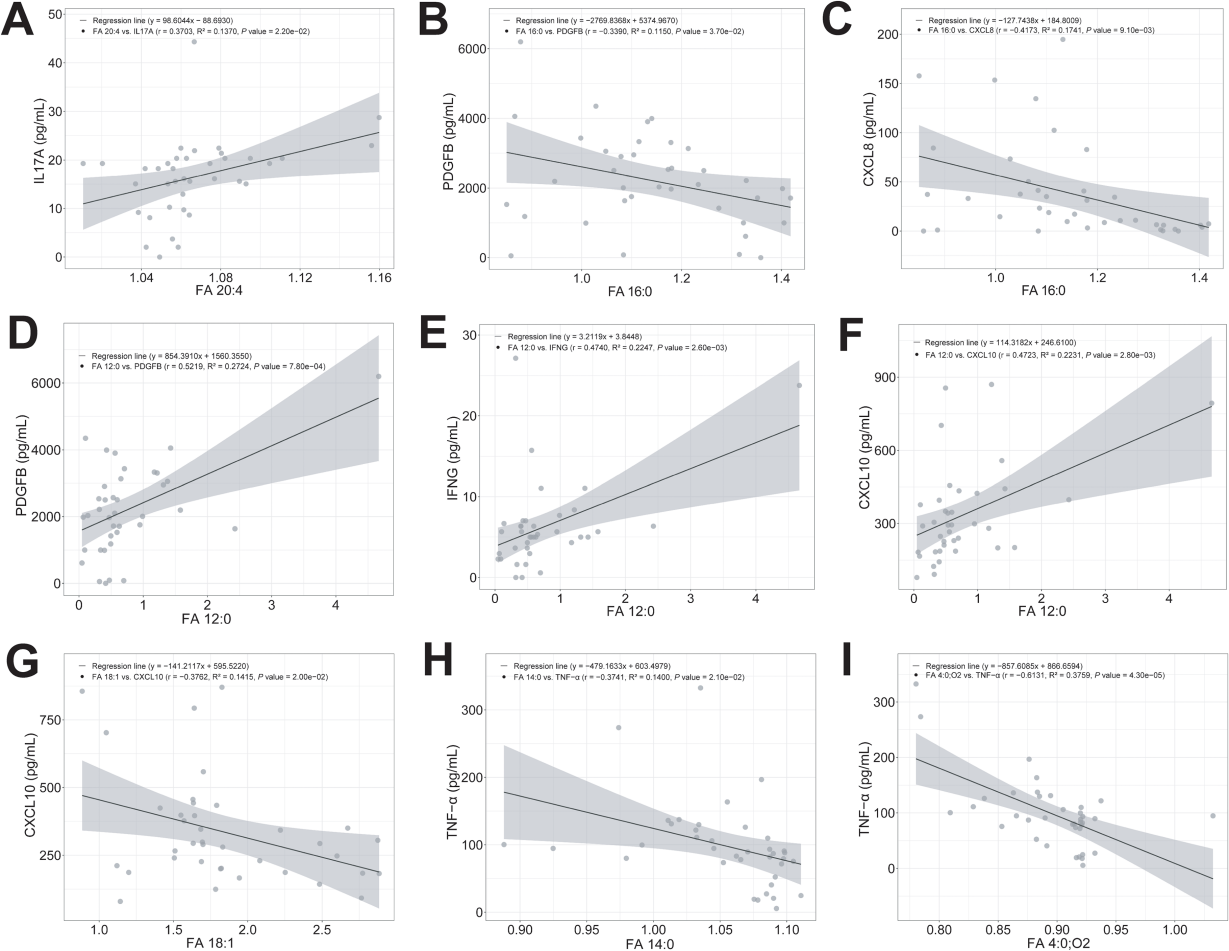
Level changes of LCFA, MCFA, and SCFA metabolites significantly correlated with cytokines and chemokines concentrations in RRMS. Correlation between the level changes of LCFA FA 20:4 and IL17A **(A)**, LCFA FA 16:0 and PDGFB, and CXCL8 **(B, C)**, MCFA FA 12:0 and PDGFB, IFNG, and CXCL10 **(D-F)**, LCFA FA 18:1 and CXCL10 **(G)**, LCFA FA 14:0 and TNF-α **(H)**, and FA 4:0;O2 and TNF-α **(I)**. Correlation analysis was performed on confounders-corrected data. Linear regression model was used to determine the correlation coefficient (r) and probability (*P*). R² values represent the proportion of variance explained. For FA 20:4 vs. IL17A: r = 0.370, R² = 0.137, indicating only 13.7% of IL17A variance was explained by FA 20:4 levels. The gray area around the straight line denoted 95% confidence interval.

To identify key node molecules in the interaction between fatty acids and immune-inflammatory responses and their correlations, we performed linear regression analysis. Results showed a significant positive correlation between increased PUFA FA 20:4 levels and elevated pro-inflammatory IL17A concentrations (r = 0.3703, R^2^ = 0.1370, *P_adj_* = 2.20e-02) (**Figure 7A**). Increased levels of the long-chain saturated fatty acid FA 16:0 showed significant negative correlations with PDGFB (r = -0.3390, R^2^ = 0.1150, *P_adj_* = 3.70e-02) and CXCL8 (r = -0.4173, R^2^ = 0.1741, *P_adj_* = 9.10e-03) concentrations (**Figures 7B, C**). Lower MCFA FA 12:0 levels showed a significant positive correlation with decreased PDGFB concentrations (r = 0.5219, R^2^ = 0.2724, *P_adj_* = 7.80e-04) (**Figure 7D**). Also, even though there were no significant differences in IFNG, CXCL10, FA 14:0, and FA 4:0;O2 levels between RRMS patients and HCs, we still observed correlations between these factors and FA 12:0, FA 18:1, and TNF-α. For example, FA 12:0 levels showed significant positive correlations with IFNG (r = 0.4740, R^2^ = 0.2247, *P_adj_* = 2.60e-03) and CXCL10 (r = 0.4723, R^2^ = 0.2231, *P_adj_* = 2.80e-03) levels (**Figures 7E, F**). FA 18:1 levels showed a significant negative correlation with CXCL10 levels (r = -0.3762, R^2^ = 0.1415, *P_adj_* = 2.00e-02) (**Figure 7G**). TNF-α levels showed significant negative correlations with FA 14:0 (r = -0.3741, R^2^ = 0.1400, *P_adj_* = 2.10e-02) and FA 4:0;O2 (r = -0.6131, R^2^ = 0.3759, *P_adj_* = 4.30e-05) levels (**Figures 7H, I**). While statistically significant, these correlations were weak to moderate (r = 0.30-0.40, R² = 0.11-0.14), suggesting that fatty acids alone explain only a modest proportion of cytokine variance, and other factors predominate. Taken together, these results suggested that LCFAs like FA 20:4 and FA 16:0, as well as the MCFA FA 12:0, may play a substantial role in modulating peripheral inflammatory responses.

### 3.5 Representative LCFA metabolites might serve as potential biomarkers for predicting RRMS

**Figure 8:**
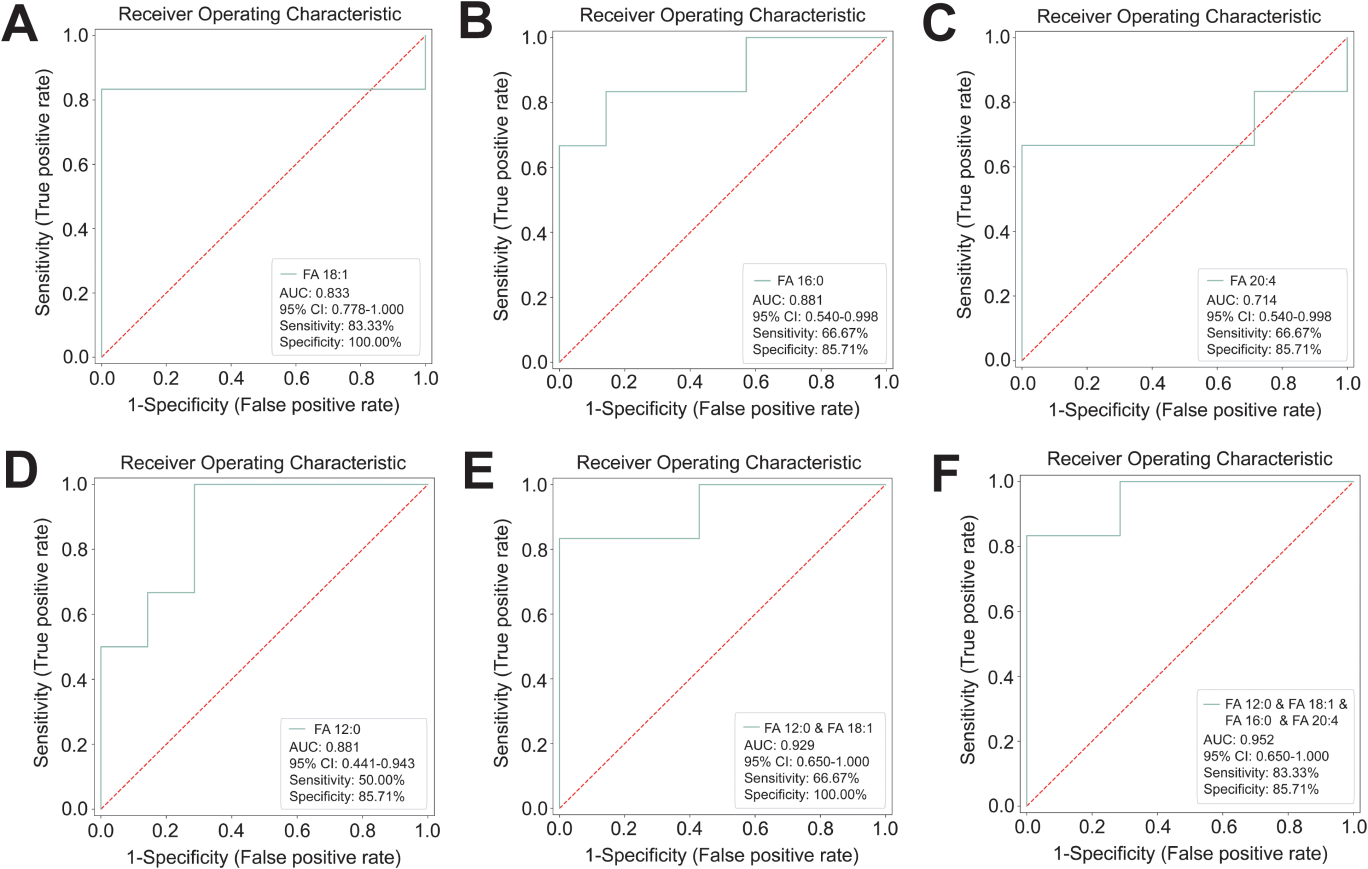
ROC curve analysis of potential LCFA biomarkers for predicting RRMS. **(A)** Receiver operating characteristic (ROC) analysis of individual LCFA metabolites such as FA 18:1 (AUC = 0.833) **(A)**, FA 16:0 (AUC = 0.881) **(B)**, and FA 20:4 (AUC = 0.714) **(C)**, and MCFA FA 12:0 (AUC = 0.881) **(D)**, and their combination such as FA 12:0 with FA 18:1 (AUC = 0.929) **(E)**, and FA 12:0, FA 18:1, FA 16:0, and FA 20:4 (AUC = 0.952) **(F)**.

To determine whether LCFAs FA 20:4, FA 18:1, FA 16:0 and MCFA FA 12:0 was able to predict RRMS, we performed receiver operating characteristic (ROC) curve analysis. ROC analysis showed that FA 18:1 (AUC = 0.833, specificity = 100.00%, sensitivity = 83.33%, 95% CI: 0.778-1.000), FA 16:0 (AUC = 0.881, specificity = 85.71%, sensitivity = 66.67%, 95% CI: 0.540-0.998), FA 20:4 (AUC = 0.714, specificity = 85.71%, sensitivity = 66.67%, 95% CI: 0.540-0.998), and FA 12:0 (AUC = 0.881, specificity = 85.71%, sensitivity = 50.00%, 95% CI: 0.441-0.943) had moderate predictive power for RRMS (**Figures 8A–D**). The predictive power for RRMS was significantly enhanced by combining more than two fatty acid metabolites, such as FA 12:0 with FA 18:1 (AUC = 0.929, specificity = 100.00%, sensitivity = 66.67%, 95% CI: 0.650-1.000) (**Figure 8E**), and the combination of FA 12:0, FA 18:1, FA 16:0, and FA 20:4 (AUC = 0.952, specificity = 85.71%, sensitivity = 83.33%, 95% CI: 0.650-1.000) (**Figure 8F**). These results indicated that LCFAs includes FA 20:4, FA 18:1, and FA 16:0 have the potential to serve as auxiliary biomarkers for predicting RRMS. While ROC analysis shows promise, the relatively wide confidence intervals reflect our sample size. The biomarker panel requires validation in larger, multicenter cohorts before clinical implementation.

## 4 Discussion

Our plasma lipidomic analysis of Chinese RRMS patients during relapse reveals a distinct pattern of LCFA dysregulation characterized by elevated palmitic acid (FA 16:0), oleic acid (FA 18:1), and arachidonic acid (FA 20:4), coupled with reduced dodecanoic acid (FA 12:0). These findings extend prior metabolomic studies in MS while highlighting unique aspects of LCFA metabolism in Chinese cohorts. In serum lipidomic analyses of predominantly Caucasian RRMS patients, Shi et al. identified elevated lysophosphatidyl-cholines, sphingomyelins, and acylcarnitines, but did not report specific LCFA alterations, suggesting our targeted LCFA approach captures complementary aspects of lipid pathology (47). Conversely, Amatruda et al. found decreased lysophosphatidic acid 18:2 (LPA 18:2) in progressive MS patients with rapid deterioration, contrasting with our observation of increased arachidonic acid in relapsing disease—potentially reflecting fundamental metabolic differences between progressive and relapsing-remitting stages (50). Notably, our findings differ from Penkert et al.’s twin study which reported decreased ether phospholipids (PC O-, PE O-) in largely stable MS patients, suggesting that LCFA elevations may be more dynamic during acute relapse episodes (48). Furthermore, Schoeps et al. demonstrated that phosphatidylcholines predict neurofilament light chain (NfL) levels while PUFAs (including arachidonic acid) are protective against subsequent disease activity in pediatric MS (49); our correlation of arachidonic acid with IL-17A provides mechanistic insight into this paradox, suggesting that PUFAs’ metabolic state during active inflammation may drive Th17-mediated responses despite their anti-inflammatory potential. The elevated LCFAs might originate from active white matter lesion degradation products crossing the blood-brain barrier, consistent with Ladakis et al.’s observation of altered brain tissue lipid metabolism in MS lesions (51). Our ROC analysis demonstrating high discriminatory power for LCFA combinations (AUC 0.952) complements prior biomarker models and may offer a relapse-specific signature. However, our modest sample size and peripheral-only sampling, limitations shared with previous plasma-based studies, necessitate validation in larger multi-ethnic cohorts integrating central and peripheral lipidomic measures.

Numerous studies have indicated that metabolomics and immune system crosstalk was crucial for regulating autoimmune responses and driving cancer and neurodegenerative disease pathogenesis (74–79). For example, sphingolipid metabolites were key regulators of T cell-specific differentiation and astrocyte immune responses in inflammatory diseases like MS (80–88). Recent studies showed that SCFA propionic acid increases the number of Tregs and decreases the amount of Th1 and Th17 cells in MS patients, thus reversing Treg/Th17 imbalance and improving MS progression (89). Independent studies indicated that butyric and propionic acids boost gut-associated Tregs, significantly alleviating immune-inflammatory responses and MS progression (90–96). However, research on the metabolic changes of LCFAs in relapsed Chinese patients with RRMS and their potential pathogenic mechanisms in MS has been lacking. This study focuses on peripheral LCFAs metabolic abnormalities and their latent interplay with immuno-inflammatory responses during relapses in RRMS-affected Chinese patients.

In this study, we found significant changes in plasma fatty acid metabolism in RRMS patients compared to HCs, with elevated levels of LCFAs like FA 16:0, FA 18:1, and FA 20:4 (**Figure 3B**), aligning with earlier studies (97–99). Conversely, MCFA FA 12:0 levels were reduced in RRMS samples (**Figure 3B**). Additionally, phospholipids like glycerophosphocholine and sphingolipids like sphingosine 1-phosphate were also decreased in RRMS samples compared to HCs (**Figures 2A, B**). It’s well established that MS was characterized by demyelination, with lipids like FA 18:1 being crucial for myelin formation. Early studies indicated that the brain white matter of MS patients is mainly composed of FA 18:1, FA 16:0, FA 18:0, and FA 20:4 (97). The origin of elevated plasma LCFAs in RRMS remains unclear. Several non-mutually exclusive possibilities exist: (a) peripheral metabolic dysregulation due to systemic inflammation; (b) spillover from CNS demyelination, though we have no direct evidence; or (c) altered hepatic lipid metabolism secondary to inflammatory cytokines. Distinguishing these mechanisms would require paired CSF and plasma studies, as well as isotopic tracing experiments, which are beyond the scope of this work. While the fold change for FA 20:4 was modest (2% increase), its biological relevance may be multifaceted: (a) small but consistent alterations in precursor pools can have amplified effects on downstream eicosanoid signaling cascades; (b) plasma levels may not fully reflect tissue-specific changes in the CNS; and (c) the statistical significance likely reflects high analytical precision rather than clinical impact. We therefore interpret this finding cautiously and emphasize the need for functional validation. Besides, we acknowledge that statistically significant changes with small effect sizes may have limited clinical utility as standalone biomarkers but may represent important pathophysiological signals worth investigating in mechanistic studies.

These results suggested the potential of these LCFAs as auxiliary diagnostic biomarkers for RRMS. ROC analysis showed that FA 18:1 (AUC = 0.833, specificity = 100.00%, sensitivity = 83.33%, 95% CI: 0.778-1.000), FA 16:0 (AUC = 0.881, specificity = 85.71%, sensitivity = 66.67%, 95% CI: 0.540-0.998), and FA 20:4 (AUC = 0.714, specificity = 85.71%, sensitivity = 66.67%, 95% CI: 0.540-0.998) have moderate predictive power for RRMS (**Figures 8A–C**). The predictive power significantly improved with combinations like FA 12:0 with FA 18:1 (AUC = 0.929, specificity = 100.00%, sensitivity = 66.67%, 95% CI: 0.650-1.000) and the combination of FA 12:0, FA 18:1, FA 16:0, and FA 20:4 (AUC = 0.952, specificity = 85.71%, sensitivity = 83.33%, 95% CI: 0.650-1.000) (**Figures 8E, F**). These results indicated that plasma LCFA FA 16:0, FA 18:1, and FA 20:4 may serve as auxiliary diagnostic biomarkers for RRMS. However, it remains unclear whether the metabolic alterations of FA 16:0, FA 18:1, and FA 20:4 are merely products of CNS lesion during MS relapses or if they also contribute to RRMS onset and progression.

In addition, in the relapse phase of RRMS patients, plasma levels of the pro-inflammatory cytokines TNF-α and IL17A were significantly elevated, while levels of pro-inflammatory chemokines (e.g., CCL3, CCL4, and CCL5) were markedly reduced (**Figure 4**). These results indicated an enhanced peripheral immune-inflammatory response mediated by TNF-α and IL17A during RRMS relapses. Recent study indicated a significant increase in Th17 cells and a marked decrease in Tregs in MS patients, leading to an imbalance between Th17 and Tregs cells (89). We speculated that these concentration changes in cytokines and chemokines may stem from the imbalance of Th1/Th17 and Tregs cells during MS relapses. Specifically, activated CD4^+^ and CD8^+^ T cells, as well as Th17 cells, produce and release TNF-α and IL17A. The significantly increased IL17A and TNF-α further suppress Tregs function and block the release of chemokines such as CCL3, CCL4, and CCL5. Notably, we observed modest interactions between LCFAs (e.g., FA 16:0, FA 18:1, FA 20:4) and immune-inflammatory responses mediated by factors such as IL17A, CCL11, CXCL10, and PDGFB (**Figures 6A, B**). Also, we observed a modest, statistically significant correlation between specific LCFA metabolite and inflammatory mediators (**Figure 7**). For instance, FA 20:4 correlated with IL17A (r = 0.370, *P_adj_* < 0.05) (**Figure 7A**), and FA 16:0 correlated with PDGFB (r = -0.339, *P_adj_* < 0.05) (**Figure 7B**). However, whether these upregulated LCFAs (e.g., FA 16:0, FA 18:1, FA 20:4) regulate the migration of peripheral CD4^+^ and CD8^+^ T cells to the CNS and the immune-inflammatory responses mediated by Th17 and Tregs cells, thereby influencing MS relapses and progression, remains unclear.

However, the current study had several limitations. First, the sample size of RRMS patients and HCs recruited in this study is relatively small. The high R² intercepts in permutation testing (R² = 0.76) suggest possible overfitting of the OPLS-DA model, likely due to our relatively small sample size relative to the number of metabolic features. While the negative Q² intercept (-0.23) confirms the model’s predictive validity exceeds chance, we acknowledge that larger sample sizes or additional regularization techniques (e.g., sparse OPLS-DA) would improve model robustness. This overfitting concern reinforces the necessity of external validation before any clinical application. A significant limitation is the lack of external validation cohort. While cross-validation was performed, the relatively wide confidence intervals reflect our modest sample size. Thus, definitive clinical utility requires completion of our ongoing prospective validation study and comparison with established biomarkers such as NfL. In future research, it is necessary to further validate the association of LCFAs such as FA 16:0, FA 18:1, and FA 20:4 with RRMS in an expanded sample population, and explore their potential clinical significance. Second, we only detected the metabolic changes of LCFAs and immune-inflammatory responses in the peripheral system of RRMS patients, without delving into the metabolic state of LCFAs in active CNS lesion areas and their crosstalk with immune responses. This limited the comprehensive reflection of pathological activities during MS relapses. In future studies, we will further analyze RRMS cerebrospinal fluid or postmortem brain tissues to better understand LCFAs metabolic abnormalities and their relationship with immune-inflammatory responses in the relapse stage. Third, the correlations between LCFAs (e.g., FA 20:4 and FA 16:0) and inflammatory cytokines, while statistically significant after FDR correction, are weak-to-moderate in strength (r = 0.33-0.47, R² = 0.12-0.23). This indicates that plasma lipid levels explain only a modest proportion of cytokine variance, with the majority of inflammatory activity driven by other factors (such as genetic, cellular, environmental factor). We caution against over-interpreting these associations as evidence of strong metabolic-immune coupling without functional validation. In future study, we will conduct *in vitro* experiments on specific LCFA (e.g., FA 20:4) modulating immune-inflammatory reactions of T cells (e.g., CD4^+^ and CD8^+^ T cells, or Tregs) and neuroglial cells, and LCFA intervention of in EAE models. This will demonstrate that LCFA such as FA 20:4 is capable of regulating peripheral and CNS inflammation, promoting RRMS relapses and progression, rather than just being products of RRMS pathological events. Fourth, the current study lacks dietary assessment, which is a significant confounder in lipidomic studies. Future studies must include detailed dietary questionnaires or food diaries to control for this variable. Additionally, we have also acknowledged that gut microbiome composition, which affects fatty acid metabolism, was not assessed and should be included in future studies. Fifth, multiple testing represents a significant limitation. Although we controlled the false discovery rate within each analysis type using FDR correction, there is still an inherent risk of false positive findings. The exploratory nature of this study prioritizes hypothesis generation over definitive conclusions, but readers should interpret findings with appropriate caution. Future confirmatory studies should pre-specify a limited number of primary hypotheses and apply more stringent correction (e.g., Bonferroni or hierarchical FDR). Sixth, our study provides limited granularity regarding clinical state beyond MRI-confirmed relapse. We lacked systematic measures of relapse severity, symptom-specific metabolic signatures, and prospective EDSS change from baseline. While exploratory analysis suggests potential associations between RRMS relapse symptom and fatty acid profiles, our sample size is insufficient to draw definitive conclusions. Future studies should incorporate detailed clinical rating scales (e.g., MSSS, MSFC) and longitudinal follow-up to correlate metabolic signatures with specific clinical trajectories. Seventh, our cohort represents a specific subset of RRMS patients with prior DMT exposure followed by prolonged treatment interruption—a pattern more common in resource-limited settings but not representative of continuously treated populations. This distinguishes our findings from studies of treatment-naïve RRMS at diagnosis or chronic treated cohorts. The metabolic signatures we observed may reflect both: (a) underlying disease pathophysiology, and (b) consequences of prolonged immunomodulatory withdrawal. Therefore, generalizability to continuously treated patients or newly diagnosed cohorts is limited and requires specific validation. Eighth, we acknowledge that many observed metabolic and inflammatory changes may not be specific to RRMS. Elevated IL17A, TNF-α, and altered PUFA metabolism are common to numerous autoimmune diseases, including SLE, RA, and NMOSD. Our study design (relapse-phase, treatment-naïve RRMS) cannot definitively distinguish whether LCFA alterations represent: (a) RRMS-specific pathophysiology, (b) general inflammatory responses common to autoimmune diseases, or (c) consequences of prolonged DMT interruption. To address this critical limitation, future studies must include disease comparator groups (e.g., NMOSD, SLE) with matched inflammatory status and treatment history. The clinical utility of our LCFA panel may therefore lie not in RRMS-specific diagnosis, but in monitoring inflammatory activity across Th17-mediated autoimmune diseases, which we are currently exploring in a multi-disease validation cohort. While our LCFA panel may lack absolute specificity for RRMS, its potential clinical utility lies in complementing existing tools rather than replacing them. Similar to how CRP monitors inflammatory activity across multiple conditions, LCFA signatures could track autoimmune disease activity, particularly in Th17-mediated disorders like RRMS, NMOSD, and SLE. The LCFA panel’s greatest value may be in: (a) frequent, non-invasive monitoring when MRI is contraindicated; (b) pharmacodynamic assessment of emerging lipid-targeting therapies; and (c) prognostic stratification to guide treatment intensity.

## 5 Conclusion

This observational study reveals altered plasma LCFA profiles in treatment-naïve Chinese RRMS patients during relapse, with modest but statistically significant correlations with pro-inflammatory cytokines/chemokines. These findings generate testable hypotheses about lipid-immune crosstalk in MS pathophysiology but do not establish causation. The identified LCFA panel requires validation in independent cohorts and different disease stages before clinical utility as biomarkers can be claimed. Future mechanistic studies are needed to determine whether these lipid changes are drivers or consequences of neuroinflammation.

## Data Availability

The metabolomics data have been deposited to MetaboLights (100) repository with the study identifier MTBLS13949.

## Supplementary material

Supplementary Figure 1 Concentration changes of sixteen representative DAMs in the RRMS and HC samples. **(A-G)** Box plots showed the abundances of seven up-regulated DAMs, including FA 20:4, FA 18:1, FA 16:0, L-Glutamic acid, Biotin, Retinoyl b-glucuronide, and Deoxyadenosine, in RRMS and HC samples. **(H-P)** Box plots showed the contents of nine down-regulated DAMs, including FA 12:0, 5-Aminopentanoic acid, Homovanillic acid, Uridine, L-Tyrosine, L-Methionine, L-Arogenate, N-Acetyl-L-Aspartic acid, and trans-Cinnamate, in RRMS and HC samples. The normalized intensity values on the y-axis were Log_2_ transformed. Samples between the two groups were compared using independent t-tests. *P* values were FDR-corrected using BH method. *: *P_adj_* < 0.05, **: *P_adj_* < 0.01, ***: *P_adj_* < 0.001.

Supplementary Figure 2 Pearson correlation analysis between the level changes of fatty acids and cytokines within RRMS and HC group. **(A)** Heatmap showed fifteen significant correlations between the concentration changes of fourteen fatty acids and sixteen cytokines/chemokines within RRMS group. **(B)** Heatmap presented five significant correlations between the level changes of fourteen fatty acids and sixteen cytokines/chemokines within HC group. Color intensity reflects correlation coefficient magnitude, red and green presents positive and negative correlation, respectively; only *P* < 0.05 correlations are highlighted with asterisks. *: *P* < 0.05, **: *P* < 0.01, ***: *P* < 0.001.

## Glossary

AUC: area under the curve
BH: Benjamini-Hochberg
BMI: body mass index
CCL2: C-C motif chemokine ligand 2
CNS: central nervous systems
CRP: C-reactive protein
CSF: cerebrospinal fluid
CXCL8: C-X-C motif chemokine ligand 8
DAM: differentially abundant metabolite
EAE: experimental autoimmune encephalomyelitis
EDSS: expanded disability severity scale
FDR: false discovery rate
HC: healthy control
HPLC: high-performance liquid chromatography
IFNG: Interferon γ
IL17A: interleukin 17A
KEGG: Kyoto Encyclopedia of Genes and Genomes
LCFA: long-chain fatty acid
LC-MS/MS: liquid chromatography-tandem mass spectrometry
MCFA: medium-chain fatty acid
MS: multiple sclerosis
MSFC: multiple sclerosis functional composite
MSSS: multiple sclerosis severity scale
MUFA: mono-unsaturated fatty acid
NMOSD: neuromyelitis optica spectrum disorder
OPLS-DA: orthogonal partial least squares discriminant analysis
PCA: principal component analysis
PDGFB: platelet derived growth factor subunit B
PLS-DA: partial least squares discriminant analysis
PPMS: primary progressive multiple sclerosis
PUFA: poly-unsaturated fatty acid
RA: rheumatoid arthritis
ROC: receiver operator characteristic
RRMS: relapsing-remitting multiple sclerosis
SCFA: short-chain fatty acid
SLE: systemic lupus erythematosus
Th1/17: T helper type 1/17 cells
TNF-α: tumor necrosis factor-α
Tregs: regulatory T cells
VIP: importance for the projection
