# Supplementary figures and images for: Aberrant long-chain fatty acids metabolism and its interplay with immuno-inflammatory responses in relapsing-remitting multiple sclerosis
Source: Front Immunol. 2026 Mar 24;17:1766322. doi: 10.3389/fimmu.2026.1766322 (PMC13053256; doi:10.3389/fimmu.2026.1766322)

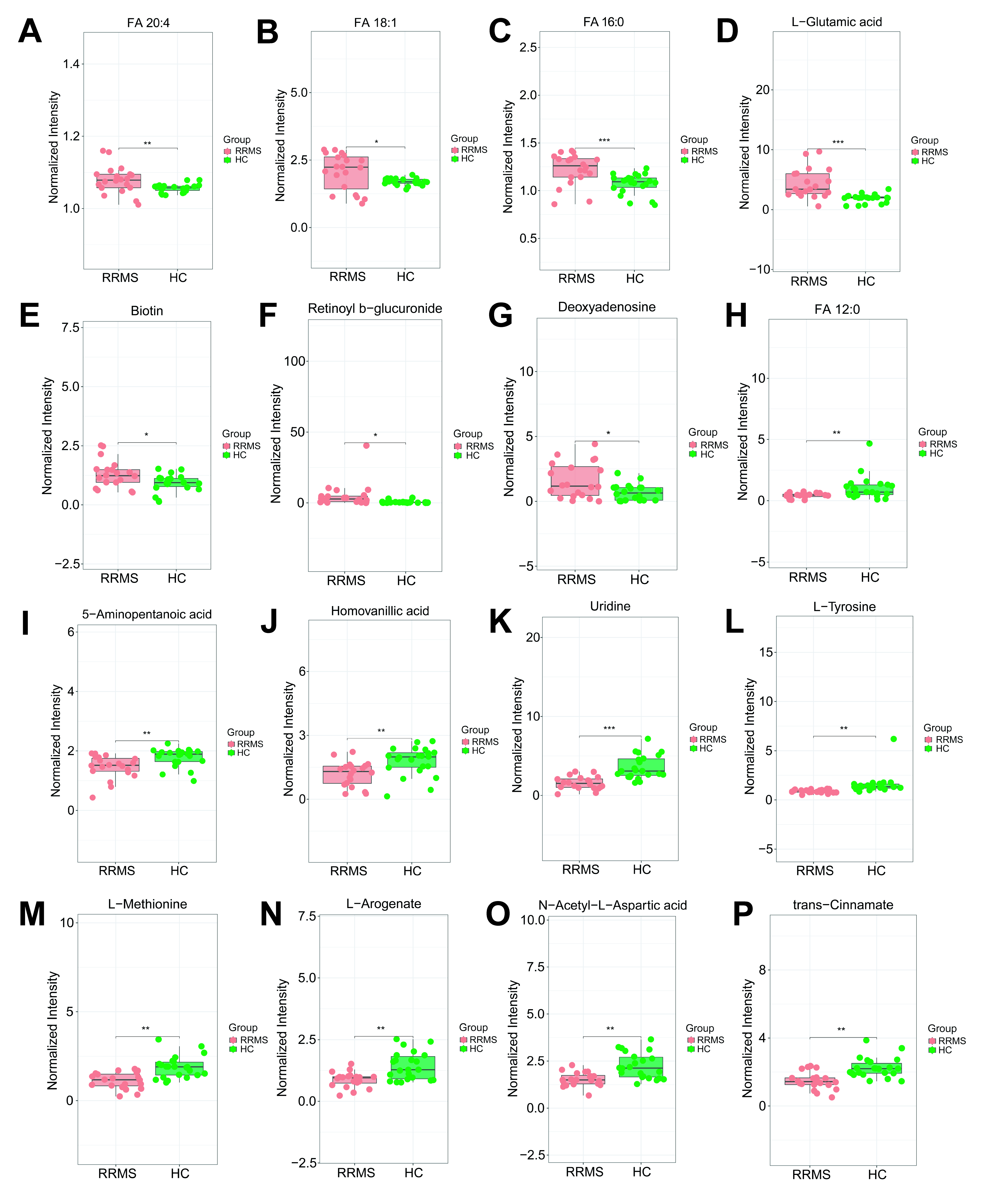

Supplement: Supplementary Figure 1 — Concentration changes of sixteen representative DAMs in the RRMS and HC samples. (A-G) Box plots showed the abundances of seven up-regulated DAMs, including FA 20:4, FA 18:1, FA 16:0, L-Glutamic acid, Biotin, Retinoyl b-glucuronide, and Deoxyadenosine, in RRMS and HC samples. (H-P) Box plots showed the contents of nine down-regulated DAMs, including FA 12:0, 5-Aminopentanoic acid, Homovanillic acid, Uridine, L-Tyrosine, L-Methionine, L-Arogenate, N-Acetyl-L-Aspartic acid, and trans-Cinnamate, in RRMS and HC samples. The normalized intensity values on the y-axis were Log2 transformed. Samples between the two groups were compared using independent t-tests. P values were FDR-corrected using BH method. *: Padj < 0.05, **: Padj < 0.01, ***: Padj < 0.001. [file Image1.tif]

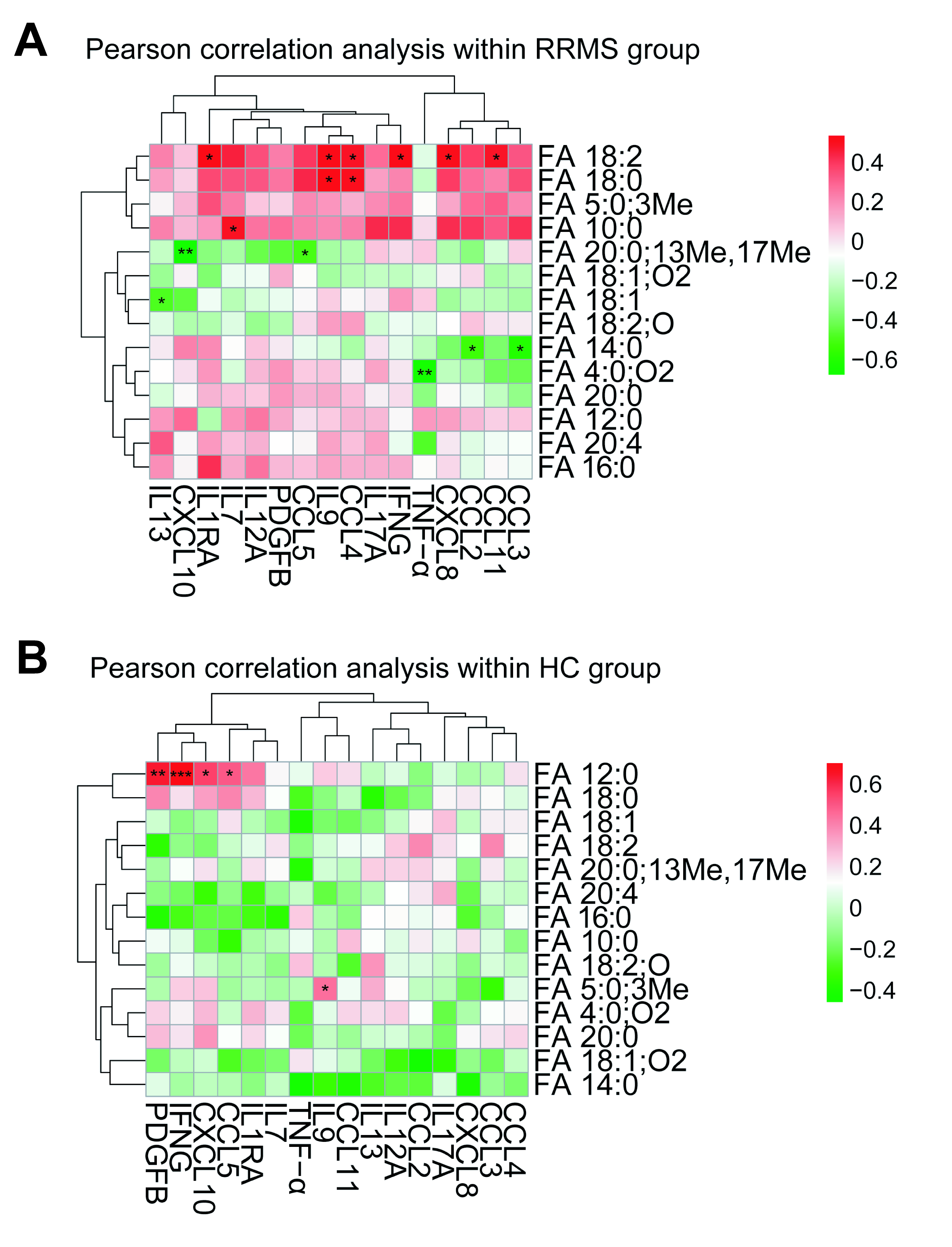

Supplement: Supplementary Figure 2 — Pearson correlation analysis between the level changes of fatty acids and cytokines within RRMS and HC group. (A) Heatmap showed fifteen significant correlations between the concentration changes of fourteen fatty acids and sixteen cytokines/chemokines within RRMS group. (B) Heatmap presented five significant correlations between the level changes of fourteen fatty acids and sixteen cytokines/chemokines within HC group. Color intensity reflects correlation coefficient magnitude, red and green presents positive and negative correlation, respectively; only P < 0.05 correlations are highlighted with asterisks. *: P < 0.05, **: P < 0.01, ***: P < 0.001. [file Image2.tif]
