# Supplementary material for: Aberrant long-chain fatty acids metabolism and its interplay with immuno-inflammatory responses in relapsing-remitting multiple sclerosis
Source: Front Immunol. 2026 Mar 24;17:1766322. doi: 10.3389/fimmu.2026.1766322 (PMC13053256; doi:10.3389/fimmu.2026.1766322)
Supplement: Supplementary file 3 [file Table1.docx]

**Table S1** Class abbreviations for category fatty acids identified in this study.

| **Class abbreviation** | **Fatty acids category** |
| --- | --- |
| FA01 | Fatty acids and conjugates |
| FA02 | Fatty amides |
| FA03 | Fatty amides (including N-acyl amines and N-acyl ethanolamines) |
| PR01 | Isoprenoids |
| SP01 | Sphingoid bases |
| ST01 | Sterols |
| ST02 | Steryl esters |
| ST03 | Steryl glycosides |
| ST04 | Bile acids and derivatives |
| ST05 | Acyl steryl glycosides |
