# Supplementary material for: Aberrant long-chain fatty acids metabolism and its interplay with immuno-inflammatory responses in relapsing-remitting multiple sclerosis
Source: Front Immunol. 2026 Mar 24;17:1766322. doi: 10.3389/fimmu.2026.1766322 (PMC13053256; doi:10.3389/fimmu.2026.1766322)
Supplement: Supplementary file 4 [file Table2.docx]

**Table S2** Shorthand notation of 14 fatty acids and its derivatives identified in this study.

| **Fatty acids and derivatives** | **Shorthand notation** |
| --- | --- |
| Linoleic acid | FA 18:2 |
| α-dimorphecolic acid | FA 18:2;O |
| Capric acid | FA 10:0 |
| 12,13-DiHOME | FA 18:1;O2 |
| Dodecanoic acid | FA 12:0 |
| Isovaleric acid | FA 5:0;3Me |
| Succinic acid | FA 4:0;O2 |
| Myristic acid | FA 14:0 |
| Stearic acid | FA 18:0 |
| Arachidic acid | FA 20:0 |
| Arachidonic acid | FA 20:4 |
| Palmitic acid | FA 16:0 |
| Oleic acid | FA 18:1 |
| Bovinic acid | FA 20:0;13Me,17Me |

Note: The notation follows the format FA (fatty acyl) followed by carbon atoms:double bond equivalents. Additional structural features are denoted after a semicolon: O indicates hydroxy groups (e.g., ;O for monohydroxy, ;O2 for dihydroxy/dicarboxylic), and Me indicates methyl branches with positional numbering.
