# Supplementary material for: Aberrant long-chain fatty acids metabolism and its interplay with immuno-inflammatory responses in relapsing-remitting multiple sclerosis
Source: Front Immunol. 2026 Mar 24;17:1766322. doi: 10.3389/fimmu.2026.1766322 (PMC13053256; doi:10.3389/fimmu.2026.1766322)
Supplement: Supplementary file 5 [file Table3.docx]

**Table S3** Cohen’s d value for 16 cytokines and chemokines.

| **Cytokine/Chemokine** | **Cohen’s d** | **Interpretation** |
| --- | --- | --- |
| TNF-α | -1.415 | Very large |
| IL17A | -1.268 | Very large |
| CXCL8 | 1.709 | Very large |
| CCL4 | 1.368 | Very large |
| CCL3 | 1.326 | Very large |
| CCL2 | 1.325 | Very large |
| IL12A | 1.284 | Very large |
| PDGFB | 1.212 | Very large |
| CCL5 | 1.076 | Large |
| IL7 | 0.898 | Large |
| IL1RA | 0.894 | Large |
| IL9 | 0.888 | Large |
| IFNG | 0.640 | Medium |
| CXCL10 | 0.580 | Medium |
| CCL11 | 0.287 | Small |
| IL13 | 0.142 | Negligible |
